# Supplementary material for: Cognitive and mental fatigue in chronic pain: cognitive functions, emotional aspects, biomarkers and neuronal correlates—protocol for a descriptive cross-sectional study
Source: BMJ Open. 2023 Mar 29;13(3):e068011. doi: 10.1136/bmjopen-2022-068011 (PMC10069545; doi:10.1136/bmjopen-2022-068011)
Supplement: Author's manuscript [file bmjopen-2022-068011.draft_revisions.pdf]

BMJ Open

BMJ Open is committed to open peer review. As part of this commitment we make the peer review history of every article we publish publicly available.

When an article is published we post the peer reviewers' comments and the authors' responses online. We also post the versions of the paper that were used during peer review. These are the versions that the peer review comments apply to.

The versions of the paper that follow are the versions that were submitted during the peer review process. They are not the versions of record or the final published versions. They should not be cited or distributed as the published version of this manuscript.

BMJ Open is an open access journal and the full, final, typeset and author-corrected version of record of the manuscript is available on our site with no access controls, subscription charges or pay-per-view fees (http://bmjopen.bmj.com).

If you have any questions on BMJ Open's open peer review process please email info.bmjopen@bmj.com

BMJ Open

Fatigue in chronic pain—cognitive functions, emotional aspects, biomarkers and neuronal correlates: protocol for a descriptive cross-sectional study

Journal:	*BMJ Open*
Manuscript ID	bmjopen-2022-068011
Article Type:	Protocol
Date Submitted by the Author:	06-Sep-2022
Complete List of Authors:	Möller, Marika; Karolinska Institutet, Clinical Sciences; Danderyd University Hospital,<br>Berginstrom, Nils; Umea Universitet, Psychology; Umea Universitet, Community Medicine and Rehabilitation, Rehabilitation Medicine<br>Ghafouri, B; Linköping University,<br>Holmqvist, Anna; Karolinska Institutet, Clinical Sciences; Danderyd University Hospital,<br>Lofgren, Monika; Karolinska Institutet; Danderyd University Hospital,<br>Nordin, Love; Karolinska Institutet, Department of Neurobiology, Caring Sciences and Society<br>Stålnacke, Britt-Marie; Umea Universitet, Department of Community Medicine and Rehabilitation, Rehabilitation Medicine
Keywords:	PAIN MANAGEMENT, RADIOLOGY & IMAGING, NEUROLOGY

SCHOLARONE™
Manuscripts

Fatigue in chronic pain—cognitive functions, emotional aspects, biomarkers and neuronal correlates: protocol for a descriptive cross-sectional study

*Möller, Marika C. [1,2], Berginström, Nils[3,4], Ghafouri, Bijar[5], Holmqvist, Anna[1,2], Löfgren, Monika[1,2], Nordin, Love[6], Stålnacke, Britt-Marie[3]

1. Department of Clinical Sciences, Karolinska Institutet, Stockholm, Sweden

2. Department of Rehabilitation Medicine, Stockholm, Danderyd University Hospital, Stockholm, Sweden

3. Department of Community Medicine and Rehabilitation, Rehabilitation Medicine, Umeå University, Umeå

4. Department of Psychology, Umeå University, Umeå

5. Pain and Rehabilitation Centre, and Department of Health, Medicine and Caring Sciences, Linköping University, Linköping, Sweden

6. Department of Neurobiology, Caring Sciences and Society, Karolinska Institutet, Stockholm, Sweden

* Corresponding author

Marika C. Möller (Orchid id: https://orcid.org/0000-0001-8700-5186)

Department of Rehabilitation Medicine

Danderyd University Hospital

Entrévägen 8

182 88 Stockholm

e-mail: marika.moller@ki.se

tfn: +46 8 12358555

Word count: Abstract: 291, manuscript: 3996
Data statement
Marika Möller

For peer review only

ARTICLE SUMMARY

STRENGTHS AND LIMITATIONS OF THIS STUDY

- A large cross-sectional multicenter study including 200 patients and 36 controls.
- Studying fatigue in chronic pain with objective neuropsychological measurements as well as biomarkers and brain imaging is an innovative approach to find underlying pathophysiological mechanisms in this condition.
- The patients are consecutive clinical patients currently being evaluated for pain rehabilitation, which means that the results from the study are clinically generalizable.
- In a longer perspective the study has potential to give rise to adapted or new rehabilitation methods for this patient group.
- A study limitation is a relatively small control group.

For peer review only

For peer review only - http://bmjopen.bmj.com/site/about/guidelines.xhtml

ABSTRACT

Chronic pain (CP) is one of the most frequently presenting symptoms in health care and many of them report mental fatigue and decline in cognitive functioning. However, the underlying mechanisms behind fatigue and cognitive dysfunctions in CP are still unknown.

Methods and analysis This study protocol describes a cross-sectional study aimed at investigating the presence of self-rated mental fatigue, objectively measured cognitive fatigability, and executive functions their relation to other cognitive functions, inflammatory biomarkers and brain connectivity in patients with CP. We will control for pain related factors such as pain intensity and secondary factors such as sleep disturbances and psychological well-being. Two hundred patients between 18 - 50 years with CP will be recruited for a neuropsychological investigation. The patients are compared with 36 healthy controls. Of these 36 patients and 36 controls will undergo blood sampling for inflammatory markers and of these 24 female patients and 22 female controls, between 18 – 45 years, will also undergo an fMRI investigation.

Primary outcomes are cognitive fatigability, executive inhibition, imaging, and inflammatory markers. Secondary outcomes include self-rated fatigue, verbal fluency, and working memory. The study provides an approach to study fatigue and cognitive functions in CP with objective measurements and may demonstrate new models of fatigue and cognition in CP.

Ethics and dissemination The study has been approved by the Swedish Ethics Review Board (Dnr 2018/424-31; 2018/1235-32; 2018/2395-32; 2019-66148; 2022-02838-02). The study findings will be disseminated through publications in journals within the fields of pain, neuropsychology, and rehabilitation. Results will further be spread at relevant national and international conferences, meetings, and expert forums. The results will be shared with user organizations and its members as well as relevant policy makers.

Trial registration ClinicalTrials.gov, NCT05452915. Release date: August 29, 2022

Keywords: Pain, fatigue, cognition, imaging, inflammatory markers, executive functions, attention, processing speed

For peer review only - http://bmjopen.bmj.com/site/about/guidelines.xhtml

Pain is one of the most frequently presenting symptoms in health care. When pain has been present 3 months it's classified as chronic. In Chronic Pain (CP) the central nervous system (CNS) is affected by an imbalance in the ascendent and descendent tracts. This facilitates the pain and impedes the pain inhibition. This, in turn, leads to plastic changes in the CNS, which are shown as increased sensibility for pain and other stimuli, depression and fatigue (1-3).

Many patients with CP suffer from mental fatigue (4-6) and decline in cognitive functioning (7), which to a large extent contribute to difficulties returning to work and high sick leave rates. Previous research has primarily relied on subjective estimates on both cognitive functions and fatigue. Mental fatigue is difficult to investigate because it is subjective in nature and the definition of the concept is indistinct (8). Fatigue is usually measured with questionnaires, but when using questionnaires, the results often interfere with other psychological conditions, e.g. depression (9), sleep disturbances (10) or pain (11). As depression is common in patients with CP it is difficult to determine whether the fatigue seen in these patients is primarily related to pain or whether it is secondary to depression (5), which inevitably result in a general attitude that fatigue among patients with CP is due to sleep disorders and depression (12). However, fatigue is not only a subjective experience. It can, but need not, be linked to impaired cognitive functioning (13). In patients with acquired brain injuries and other neurological conditions, fatigue has been associated to impaired attention (14), slower processing speed (15) and fatigability (16).

It is well established from studies with self-reported data that patients with CP are often and severely bothered by cognitive impairments (7). Patients with Whiplash Associated Disorders (WAD) often report not only pain, but also concentration difficulties, fatigue, headache, vertigo and decreased life satisfaction (17). Furthermore, patients with generalized pain report problems with concentration difficulties and memory problems (18). In fibromyalgia factors such as depression, fatigue, and pain intensity contribute to the cognitive ability (19). As noted above it is important to separate self-reported symptoms from functional disabilities on objective tests since self-reports can be influenced of psychological well-being (20) and test performance on effort (16).

Impaired cognitive ability in CP has been explained with either "Limited resource theory" i.e., that the pain competes for limited attentional resources in the brain or with "Neuroplasticity theory" where it is believed that impaired cognitive ability in patients with CP is caused by stress, which in turn counteracts neuroplasticity which creates neurodegenerative processes. In CP the central nervous system is affected by an imbalance in the brain's pathway system, which leads to plastic changes in the brain (21). In particular, pain activates areas of the brain that are important for attentional functions, which may explain why many patients with pain experience attention deficits

For peer review only - http://bmjopen.bmj.com/site/about/guidelines.xhtml

For peer review only

(7). The challenges associated with CP are considered to lead to a depletion of the person's self-regulating resources (22). However, these theories are criticized, as there is a lack of knowledge about precise mechanisms and the time course in the development of CP and fatigue (23). Assessments of cognitive functions are rarely included in clinical praxis of CP conditions in Sweden. However, it cannot be ruled out that fatigue, even in the case of CP, may be related to poorer attention and slow processing speed (24). Only a few studies have used neuropsychological tests to study cognitive functions in CP - mainly in patients with fibromyalgia (7). Cognitive fatigue measured with neuropsychological tests has not been studied before in patients with CP.

To enable effective treatment, it is important to find markers that can distinguish different causes of fatigue from each other. Fatigability in cognitively demanding tasks can be such a marker (25), which has been shown in patients with mild traumatic brain injury (16), multiple scleroses (26) and hormonal conditions (27, 28). Since objective measures of fatigue have not been used in previous studies in CP, it cannot be ruled out that fatigue and cognitive impairments may have similar underlying mechanisms as in neurological conditions and that fatigability could be such a marker also in CP.

Functional MRI (fMRI) may reflect increased or decreased neuronal activity in the brain as changes in neuronal activity are linked to changes in regional blood flow (rCBF) and blood oxygenation levels (BOLD). With different fMRI techniques, it is possible to study whether discrete cerebral dysfunctions can be linked to self-perceived fatigue and cognitive fatigue/fatigability. Fatigue among patients with brain injuries has been linked to specific networks for attention in the brain, involving fronto-subcortical regions (29-31). Möller and Nordin have shown that fatigue in mild traumatic brain injury is related to altered connectivity in the brain (31) and that there are different neural networks that are related to subjective compared to objective measures of (16). Whether similar patterns can also be seen in patients with pain and fatigue needs to be investigated. Connectivity changes have also been seen in CP, and patients with different pain conditions show connectivity changes (32). These studies have not included fatigue and fatigability and not used task-fMRI in the scanner.

Chronic low-grade systemic inflammation is also thought to play a major role in the onset of CP. Studies have shown that patients with generalized pain/fibromyalgia have elevated levels of immunological biomarkers (33). Similar results have been demonstrated in patients with neuropathic pain (34). It is unclear whether patients with other types of pain also have elevated levels of these proteins. It is also unknown if there is any relationship between immunological biomarkers and other variables such as fatigue and cognition.

There is thus a need for increased knowledge about how CP can affect functions such as cognition and fatigue and the underlying pathophysiological mechanisms for this. To improve and diagnostic and prognostic models as well as develop the treatment pharmacologically and/or with cognitive

For peer review only - http://bmjopen.bmj.com/site/about/guidelines.xhtml

training, the connection between pain and underlying pathophysiological mechanisms needs to be studied further with objective markers.

Objectives

The primary aim of the study is to investigate the presence of self-rated mental fatigue, objectively measured cognitive fatigability and executive functions and how these are related to other cognitive functions, pain related factors, inflammatory biomarkers, and brain connectivity in patients with CP.

1. Do patients with CP show more pronounced self-rated mental fatigue and cognitive fatigue compared to healthy controls and are fatigue measures related to cognitive functions such as process speed and attention functions?
2. What is the relationship between cognitive impairments, mental fatigue, or fatigability and
    a. the duration of pain
    b. generalization or intensity of pain

   and are they affected by covariates, such as sleep disorders and degree of depression/anxiety?
3. Is there an association between immunological biomarkers and cognitive functions/mental fatigue/cognitive fatigability?
4. Does the connectivity in the brain of patients with CP differ from healthy controls at rest as well as during activity while performing a vigilance task?
5. Is there an association between connectivity in the brain and
    a. immunological biomarkers
    b. fatigability and
    c. results on neuropsychological tests?

Methods

Study design

This is a cross-sectional explorative case-control study. STROBE-reporting guidelines will be used (35).

Study setting

Outpatient pain rehabilitation teams at Department of Rehabilitation Medicine at Danderyd University Hospital, Stockholm and the Department of Pain Rehabilitation, Pain Center, Umeå University Hospital, Umeå.

Patient and public involvement

Patients and/or the public were not involved in the design, or conduct, or reporting, or dissemination plans of this research.

For peer review only - http://bmjopen.bmj.com/site/about/guidelines.xhtml

Participants

Patients for neuropsychological assessment

Two hundred patients between 18 - 50 years with CP who are to the Department of Rehabilitation Medicine at Danderyd University Hospital or to the Department of Pain Rehabilitation, Pain Center at Umeå University Hospital will be included. No stratification of age will occur, but the majority of the patients will be women as they are overrepresented among those receiving rehabilitation for CP. In the analyzes, the patients are compared with 36 healthy controls.

Healthy controls

Thirty-six healthy controls will be matched on group level in terms of gender, age, and educational level. They will be recruited among hospital staff or their friends. For those who only do neuropsychological assessment and blood sampling a token of SEK 300 will be offered and for those who do the fMRI and blood sampling a token of SEK 1000 will be offered.

Participants for fMRI

Twenty-four (24) patients, women only, between 18 – 45 years with CP and who have undergone a neuropsychological assessment at the Department of Pain Rehabilitation, Pain Center at Umeå University Hospital will also undergo an fMRI investigation. They will be compared with 22 female controls from the healthy control group.

Participants for blood sampling

Of the included participants 36 patients and 36 controls will undergo blood sampling for immunological markers. All the participants who undergo fMRI will take blood sampling.

Eligibility criteria

Inclusion criteria

- CP
- aged 18 - 50 years for neuropsychological assessment and 18-45 for fMRI examination
- referred to the Department of Rehabilitation Medicine at Danderyd University Hospital or to the Department of Pain Rehabilitation, Pain Center, Umeå University Hospital

Exclusion criteria

- traumatic brain injury (including concussion)
- extensive psychiatric problems or substance abuse

For peer review only - http://bmjopen.bmj.com/site/about/guidelines.xhtml

For peer review only

- previously congenital or acquired brain injury and / or does not live in their own home and who needs support in everyday life
- lack of knowledge of the Swedish language
- progressive disease
- abuse
- drugs with a strong sedative effect
- pregnancy
- for fMRI examination, patients with metal objects implanted in the body or metal chips in body parts (eg after eye damage) are excluded. Patients are excluded even if they are afraid of confined spaces and if they are left-handed.

Measurements

Background information from the Swedish Quality Register for Pain Rehabilitation (SQRP) (http://www.ucr.uu.se/nrs/). Information about medication, possible trauma and background information from the time of the injury and degree of sick leave will be obtained from the medical record.

Questionnaires from SQRP

- EuroQoL five dimensions questionnaire (EQ5D) (36)
- Hospital Anxiety and Depression Scale (HADS) (37)
- Insomnia Severity Index (ISI) (38)
- Multidimensional Pain Inventory (MPI) (39).
- Screening on physical activity by the National Board of Health and Welfare (40)

Other questionnaires

- Visual analogue scale on fatigue (VAS-f) before and after neuropsychological assessment
- Visual analogue scale on fatigue (VAS-f) and pain before and after the fMRI scanning.
- Multidimensional Fatigue Inventory-20 (MFI-20) (41)
- Pain level, experienced during the neuropsychological examination, will be rated (0-10) at the end of the session.

Neuropsychological tests

- Wechsler Adult Intelligence Scale-III (WAIS-III) Digit-Symbol-Coding (Coding) (psychomotor speed, incidental memory, and fatigability). The higher values the better results (42).
- WAIS-IV Matrix reasoning (logical reasoning and premorbid level). The higher values the better results. (43)

For peer review only - http://bmjopen.bmj.com/site/about/guidelines.xhtml

For peer review only

- WAIS-IV Digit Span (verbal attention span and working memory). The higher values the better results. (44, 45).
- Delis-Kaplan Executive Function Scale (D-KEFS) Color-Word Test and Word Fluency Test (executive functions) For Color-Word test the lower values the better results and for the Word Fluency Test the higher values the better results (46).
- Ruff 2 & 7 (visual scanning and selective attention). The higher values the better results (47).
- MapCog Spectra (frequency of attention lapses). We also measure fatiguability by comparing the results in the end of the test with the beginning of the test. The lower values the better results (48).

For peer review only

PRIMARY OUTCOME VARIABLES

Outcome variables are summarized in table 1.

Table 1: Outcome measures

**Primary outcome measures**	**Description**
**1. Fatigability from WAIS-III Coding**	The subject must fill in the blank spaces with the symbol which is paired to the number during 120 seconds. Cognitive fatigue is assessed by subtracting the number of digits produced in the first 30 seconds from the number of digits produced in the last 30 seconds during the full 120-second period. A non-ascending score (< 0) is considered an indicator of cognitive fatigue. Both the total value in the difference between the production between 0-30 seconds and 91-120 seconds are measured, and a dichotomized variable (non-ascending value) will be used.
**2. D-KEFS Color-Word Test**	Inhibition of over-learned verbal responses. The test has four conditions: 1) naming colors (red, blue or green, 2) reading color words printed in black, 3) naming the color of the color words red, blue or green write in a different color than what is written, which means inhibition of an over-learned function of reading the word; 4) repeatedly switching between naming colors and reading out the printed words as quickly as possible, while at the same time the person needs to keep track of clues that indicate rule change. Contrast scores are used to examine the performance of themore complex tasks 3 and 4 and the basic tasks 1 and 2. The faster the time, the better.
**3. Fatigability on e-prime vigilance task in the fMRI scanner**	The participants are instructed to push a button as quick as they can when a set of four zeroes appears in a red rectangle and do nothing if other numbers appeared. After each response visual feedback of the reaction time is displayed. If the participant reacts at a false stimulus or if the response time of more than 1 sec. the feedback "false answer" or "no answer" is displayed respectively. The stimuli are presented at random intervals. The results are divided into quintiles and the mean reaction time is calculated for each quintile.
**4. Task-fMRI**	BOLD signal changes during fatiguing attention task.

For peer review only - http://bmjopen.bmj.com/site/about/guidelines.xhtml

**5. Resting state fMRI**	Changes in functional connectivity after performance of a fatiguing vigilance task.
**6. Inflammatory markers**	Omics analyzes will be used in this study. Omics studies are mainly exploratory and hypothesis generating, i.e., which proteins, metabolites and lipoproteins are identified cannot be determined in advance. Exploratory targeted analyzes using panels of cytokines & chemokines and neuroinflammation will be used in combination with omics.
**Secondary outcome measures**	**Description**
**1. MFI-20**	The MFI-20 consists of five scales, based on different modes of expressing fatigue. Each scale contains four items for which the person has to indicate on a seven-point scale to what extent the particular statement applies best. 'General fatigue' includes general statements concerning a person's functioning. 'Physical fatigue' refers to the physical sensation related to the feeling of tiredness. 'Reduced activities' measures reduction in activities and 'Reduced motivation' lack of motivation. 'Mental fatigue' measures cognitive symptoms related to fatigue. Some sentences are inverted and need to be rescored. On each scales the higher values the higher fatigue.
**2. Visual Analog Scale of Fatigue**	Measurement of self-rated current fatigue level. Ranges from 0 (corresponding to no fatigue) to 10 (corresponding to the worst possible fatigue).
**3. D-KEFS Word Fluency Test**	The test measures expressive language skills, initiative and working memory and consists of three different conditions: verbal phonological flow (1) where the test person for 60 seconds produces as many unique words as possible that begins with a given letter., category flow (2) where the test person for 60 seconds per category produce as many unique words as possible in two given semantic categories and category change (3) where the test person in 60<br>seconds produce so many unique words and switch between two specified semantic categories every other time. The more words, the better the performance.
**4. WAIS-IV Digit Symbol backward repetition (Working memory)**	The test person must repeat numbers that the test leader reads out the leader reads out. The number of digits is increased by one unit every two times). The test person repeats the numbers in the same order (forward repetition) or reverse order (backward repetition). Backward repetition measures auditory working memory. Both the total number of digits and the difference between forward and backward repetition are measured.

Abbreviations: BOLD - Blood Oxygen Level Dependent; D-KEFS - Delis-Kaplan Executive Function Scale; Coding – Digit-Symbol Coding Test; fMRI - Functional magnetic resonance imaging; MFI - Multidimensional Fatigue Inventory; WAIS– Wechsler Adult Intelligence Scale

Data Collection and Management

Recruitment

The pain rehabilitation clinics at the Department of Rehabilitation Medicine, Danderyd University Hospital and the Department of Pain Rehabilitation, Pain Center at Umeå University Hospital offer assessment and evidence-based team-based multimodal rehabilitation for patients with complex CP.

For peer review only - http://bmjopen.bmj.com/site/about/guidelines.xhtml

To receive rehabilitation at the clinics, a referral is required. Information about the study is sent to the referred patients at the same time as the call for initial assessment.

At time for the team assessment, the patient is asked orally by the team nurse if the testing psychologist may contact the patient to inform about the study. The patient's consent is logged, and a log is made of the number who say yes and the number and gender and age of those who say no. No personal data other than gender will be registered on those who say no. The purpose of the log is only to be able to assess generalizability based on the proportion who have agreed to participate in the study.

The testing psychologist then calls the patients and inform them about the study and if the patient agrees, an appointment is made for neuropsychological. In connection with the assessment, the psychologist ensures that they have understood the information, inquire about whether they have further questions and collect the signed consent form. Female patients who are investigated in Umeå are asked if they want to participate in the fMRI study. The patients are assessed by an independent psychologist, who does not treat the patients clinically.

The study patients will be compared with healthy controls. Healthy controls will be recruited through advertising among hospital staff. Matching at group level will take place regarding age, gender and level of education.

Imaging

In a 3-tesla MR scanner, conventional anatomical MRI sequences will be analyzed as well as sequences for detailed anatomical assessment. We use an established measurement paradigm that has previously been used on healthy subjects (49) and patients with mild traumatic brain injury (50).

All participants are examined with fMRI during a 20-minute vigilance (reaction time) task (e-prime), where fatigue is measured by recording mean reaction time / quintile. BOLD resting-state fMRI examination is performed before and after the task. In addition, MRI protocol will include standard clinical protocols that include high-resolution T1-weighted sequences and high-resolution T2-weighted Scan (FLAIR). All structural images are reviewed by a radiologist to scan for incidental findings within the brain.

The investigation is completely non-invasive and takes about 60 minutes.

Blood sampling

Venous blood samples (10-20 ml) are collected in P100 tubes from BD diagnostic, containing a protease inhibitor cocktail that prevents protein cleavage / degradation) from each subject. The blood samples are centrifuged to remove red blood cells. The plasma fraction is transferred to a new tube, portioned and stored at -86 ° C until analysis at PAINOMICS lab in Linköping, Sweden.

For peer review only - http://bmjopen.bmj.com/site/about/guidelines.xhtml

Plasma samples will be analyzed using antibody-based methods such as multiplex technology (Meso Scale Diagnostics, Rockville, MD). Instrument, MSD Technology platform, which is a multiplex instrument, which can analyze up to 92 different substances (cytokines, chemokines, growth factors and metabolic proteins) in one go. Omics analysis consisted of proteomics, metabolomics and lipoprotein profiling will be used to identify other inflammatory markers than those analyzed by the antibody-based (targeted analysis) method.

Data Management

All data material will be recorded with a participant ID and will be unidentifiable. In the statistical processing, the participants will be coded. Code key is stored separately from data and only pseudonymized data will be shared between the two sites. De-identified data will be electronically stored on the server at the two sites and will be deleted 10 years after the project has ended. The final dataset will be available to researchers actively contributing to statistical analyses and publications. Data entry will be controlled by initial exploratory analyses, including range checks, in order to promote data quality. Regarding biological specimens the samples of blood will be centrifuged and plasma will be aliquoted and stored at the University Hospital, with special notification to the biobank. The samples will be blinded before analysis. This study has a hypothesis generating character as we use proteomic methods therefore samples (that are not thawed) will be stored for future analysis according to approved ethical application.

Sample Size

Power calculation on the primary fatigue measure the WAIS-III Coding Test from WAIS-III on: For a mean difference between patients and controls of 2.5 and standard deviation of 3.0 and alpha level of 5% (two-sided) and strength of 80%, a group of 24 participants in each group is a sufficiently large sample (51).

Confidentialty

Information on participants will be handled by health care professionals adhering to Swedish Law ensuring confidentiality and data protection by coding of individual participants' collected data. Results and data will be presented at a group level in publications, rendering identification of individual patients impossible. All data will be stored accordance with the General Data Protection Regulation (GDPR). The data for participants from Stockholm area will be stored at a password protected project server in Danderyd University Hospital and for patients from Umeå region at Umeå University. Data will not be accessed by unauthorized persons.

For peer review only

DATA ANALYZES

Descriptive statistics will be used to depict demographics, injury characteristics, results on neuropsychological tests and psychological screening instruments. Multiple linear and logistic regression will be used to detect any interaction effects. Parametric methods will be used when data is normally distributed and non-parametric methods when data is skewed or on interval level or lower. Missing data will be listed as data loss.

Analysis of fMRI data will be done with special software for MRI analysis, developed at Oxford University (http://www.fmrib.ox.ac.uk/fsl/). Statistical comparison of gray matter volume, white matter damage and blood flow at rest and in cognitive activation is done in specific analysis modules where MRI data from the different groups are compared at pixel level (voxels) with methodology specifically developed for the different MRI modalities.

Data from omics and targeted analysis will be analyzed using advanced multivariate data analysis using SIMCA-P+, that is recommended method in omics where it account for multicollinearity problems and missing data and when the numbers of variable exceed the number of observations (52).

PLANS FOR COMMUNICATING IMPORTANT PROTOCOL AMENDMENTS TO RELEVANT PARTIES

Important protocol modifications will be reported to the Ethics committee in Sweden and amendments will be made to the trial registry (Clinicaltrials.gov).

PATIENT AND PUBLIC INVOLVEMENT

Patients were not involved in formulating the research question or setting the research design for the planned study.

ETHICS AND DISSEMINATION PLANS

The study was approved by the Ethics Review Board (2018/424-31; 2018/1235-32; 2018/2395-32; 2019-66148; 2022-02838-02). All participants are provided oral and written information by the neuropsychologists (AH and NB) and will give written informed consent to participate in the study to them.

Publications are planned for journals in the fields of pain, neuropsychology, and rehabilitation. Results will further be spread at relevant conferences, national and international meetings, and

For peer review only - http://bmjopen.bmj.com/site/about/guidelines.xhtml

expert forums. The results will be shared with user organizations and its members as well as relevant policy makers.

AUTHOR CONTRIBUTIONS

MCM, ML and BMS formed the original research concept. BG, LN, NB and AH contributed to the study design within their field of expertise. MCM and BMS will coordinate the project in Stockholm and Umeå in cooperation with NB, BG, AH, ML, and LN. NB and AH will be responsible for data collection and statistical analyses of the neuropsychological and demographic data in collaboration with MCM, ML and BMS. LN will be responsible for fMRI analyses in collaboration with NB. BG will be responsible for the analyses of biomarkers. MCM has written the manuscript draft. All authors have contributed with important intellectual content to the manuscript. Regarding upcoming manuscripts, we will use authorship eligibility guidelines. We will not use professional writers.

FUNDING

This study is supported by Promobilia Foundation, grant number [A22056] the research and development fund granted by the County Council of Västerbotten [RV-928951; RV-939460], and through a regional agreement between Umeå University and Västerbotten County Council (ALF) [RV-930845; RV-940388; RV-967132]. The Department of Rehabilitation Medicine and the Karolinska Institutet, Department of Clinical Sciences, Danderyd University Hospital have also provided valuable support for the study to be feasible. The funders have no vested interest in the study and have not influenced the design.

COMPETING INTERESTS

None declared.

PATIENT CONSENT FOR PUBLICATION

Consent obtained directly from patients

REFERENCES

1. Ng SK, Urquhart DM, Fitzgerald PB, Cicuttini FM, Hussain SM, Fitzgibbon BM. The Relationship between Structural and Functional Brain Changes and Altered Emotion and Cognition in Chronic Low Back Pain: A Systematic Review of MRI and fMRI Studies. Clin J Pain. 2017.
2. Flodin P, Martinsen S, Mannerkorpi K, Lofgren M, Bileviciute-Ljungar I, Kosek E, et al. Normalization of aberrant resting state functional connectivity in fibromyalgia patients following a three month physical exercise therapy. Neuroimage Clin. 2015;9:134-9.

For peer review only - http://bmjopen.bmj.com/site/about/guidelines.xhtml

3. Marchand S. The physiology of pain mechanisms: from the periphery to the brain. Rheum Dis Clin North Am. 2008;34(2):285-309.
4. Wolfe F, Hawley DJ, Wilson K. The prevalence and meaning of fatigue in rheumatic disease. J Rheumatol. 1996;23(8):1407-17.
5. Mease P, Arnold LM, Bennett R, Boonen A, Buskila D, Carville S, et al. Fibromyalgia syndrome. J Rheumatol. 2007;34(6):1415-25.
6. Fishbain DA, Cole B, Cutler RB, Lewis J, Rosomoff HL, Fosomoff RS. Is pain fatiguing? A structured evidence-based review. Pain Med. 2003;4(1):51-62.
7. Moriarty O, McGuire BE, Finn DP. The effect of pain on cognitive function: a review of clinical and preclinical research. Prog Neurobiol. 2011;93(3):385-404.
8. Kluger BM, Krupp LB, Enoka RM. Fatigue and fatigability in neurologic illnesses: proposal for a unified taxonomy. Neurology. 2013;80(4):409-16.
9. Arnold LM. Understanding fatigue in major depressive disorder and other medical disorders. Psychosomatics. 2008;49(3):185-90.
10. Akerstedt T, Knutsson A, Westerholm P, Theorell T, Alfredsson L, Kecklund G. Mental fatigue, work and sleep. J Psychosom Res. 2004;57(5):427-33.
11. Snekkevik H, Eriksen HR, Tangen T, Chalder T, Reme SE. Fatigue and depression in sick-listed chronic low back pain patients. Pain Med. 2014;15(7):1163-70.
12. Kurtze N, Svebak S. Fatigue and patterns of pain in fibromyalgia: correlations with anxiety, depression and co-morbidity in a female county sample. Br J Med Psychol. 2001;74(Pt 4):523-37.
13. Johnson SK, Lange G, DeLuca J, Korn LR, Natelson B. The effects of fatigue on neuropsychological performance in patients with chronic fatigue syndrome, multiple sclerosis, and depression. Appl Neuropsychol. 1997;4(3):145-53.
14. Ziino C, Ponsford J. Measurement and prediction of subjective fatigue following traumatic brain injury. J Int Neuropsychol Soc. 2005;11(4):416-25.
15. Johansson B, Berglund P, Rönnbäck L. Mental fatigue and impaired information processing after mild and moderate traumatic brain injury. Brain Inj. 2009;23(13-14):1027-40.
16. Moller MC, Nordin LE, Bartfai A, Julin P, Li TQ. Fatigue and Cognitive Fatigability in Mild Traumatic Brain Injury are Correlated with Altered Neural Activity during Vigilance Test Performance. Front Neurol. 2017;8:496.
17. Coppieters I, De Pauw R, Kregel J, Malfliet A, Goubert D, Lenoir D, et al. Differences Between Women With Traumatic and Idiopathic Chronic Neck Pain and Women Without Neck Pain: Interrelationships Among Disability, Cognitive Deficits, and Central Sensitization. Phys Ther. 2017;97(3):338-53.
18. Grisart J, Van der Linden M, Masquelier E. Controlled processes and automaticity in memory functioning in fibromyalgia patients: relation with emotional distress and hypervigilance. J Clin Exp Neuropsychol. 2002;24(8):994-1009.
19. Ojeda B, Duenas M, Salazar A, Mico JA, Torres LM, Failde I. Factors Influencing Cognitive Impairment in Neuropathic and Musculoskeletal Pain and Fibromyalgia. Pain Med. 2017.
20. Lundin A, de Boussard C, Edman G, Borg J. Symptoms and disability until 3 months after mild TBI. Brain Inj. 2006;20(8):799-806.
21. Ng K, Sin , Urquhart M, Donna , Fitzgerald B, Paul , Cicuttini M, Flavia , Hussain M, Sultana , Fitzgibbon M, Bernadette. The Relationship Between Structural and Functional Brain Changes and Altered Emotion and Cognition in Chronic Low Back Pain Brain Changes: A Systematic Review of MRI and fMRI Studies. The Clinical Journal of Pain. 2018;34(3):237-61.
22. Solberg Nes L, Roach AR, Segerstrom SC. Executive Functions, Self-Regulation, and Chronic Pain: A Review. Ann Behav Med. 2009;37(2):173-83.
23. van Dartel SA, Repping-Wuts JW, van Hoogmoed D, Bleijenberg G, van Riel PL, Fransen J. Association between fatigue and pain in rheumatoid arthritis: does pain precede fatigue or does fatigue precede pain? Arthritis Care Res (Hoboken). 2013;65(6):862-9.
24. Flodin P, Martinsen S, Mannerkorpi K, Lofgren M, Bileviciute-Ljungar I, Kosek E, et al. Normalization of aberrant resting state functional connectivity in fibromyalgia patients following a three month physical exercise therapy. . Neuroimage Clin. 2015;9 134-9.
25. Kluger BM, Krupp LB, Enoka RM. Fatigue and fatigability in neurologic illnesses: proposal for a unified taxonomy. Neurology. 2013;80(4):409-16.
26. Walker LA, Berard JA, Berrigan LI, Rees LM, Freedman MS. Detecting cognitive fatigue in multiple sclerosis: method matters. J Neurol Sci. 2012;316(1-2):86-92.

For peer review only - http://bmjopen.bmj.com/site/about/guidelines.xhtml

27. Möller MC, Bartfai A, Nygren de Boussard C, Flöter Rådestad A, Calissendorff J. High rates of fatigue in newly diagnosed Graves' disease. Fatigue: Biomedicine, Health & Behavior. 2014;2(3):153-62.
28. Möller MC, Rådestad AF, von Schoultz B, Bartfai A. Effect of estrogen and testosterone replacement therapy on cognitive fatigue. Gynecol Endocrinol. 2013;29(2):173-6.
29. Cantor F. Central and peripheral fatigue: exemplified by multiple sclerosis and myasthenia gravis. Pm & R. 2010;2(5):399-405.
30. Dobryakova E, Deluca J, Genova HM, Wylie GR. Neural Correlates of Cognitive Fatigue: Cortico-Striatal Circuitry and Effort-Reward Imbalance. Journal Of The International Neuropsychological Society: JINS. 2013:1-5.
31. Nordin LE, Moller MC, Julin P, Bartfai A, Hashim F, Li TQ. Post mTBI fatigue is associated with abnormal brain functional connectivity. Sci Rep. 2016;6:21183.
32. Apkarian AV. Frontiers in Neuroscience. Human Brain Imaging Studies of Chronic Pain: Translational Opportunities. In: Kruger L, Light AR, editors. Translational Pain Research: From Mouse to Man. Boca Raton, FL: CRC Press/Taylor & Francis Llc.; 2010.
33. Hysing EB, Smith L, Thulin M, Karlsten R, Bothelius K, Gordh T. Detection of systemic inflammation in severely impaired chronic pain patients and effects of a multimodal pain rehabilitation program. Scandinavian journal of pain. 2019;19(2):235-44.
34. Backryd E, Lind AL, Thulin M, Larsson A, Gerdle B, Gordh T. High levels of cerebrospinal fluid chemokines point to the presence of neuroinflammation in peripheral neuropathic pain: a cross-sectional study of 2 cohorts of patients compared with healthy controls. Pain. 2017;158(12):2487-95.
35. von Elm E, Altman DG, Egger M, Pocock SJ, Gøtzsche PC, Vandenbroucke JP. The Strengthening the Reporting of Observational Studies in Epidemiology (STROBE) statement: guidelines for reporting observational studies. Ann Intern Med. 2007;147(8):573-7.
36. Group E. EuroQol--a new facility for the measurement of health-related quality of life. Health policy (Amsterdam, Netherlands). 1990;16(3):199-208.
37. Zigmond AS, Snaith RP. The Hospital Anxiety and Depression Scale. Acta Psychiatrica Scandinavica. 1983.
38. Bastien CH, Vallieres A, Morin CM. Validation of the Insomnia Severity Index as an outcome measure for insomnia research. Sleep Med. 2001;2(4):297-307.
39. Kerns RD, Turk DC, Rudy TE. The West Haven-Yale Multidimensional Pain Inventory (WHYMPI). Pain. 1985;23(4):345-56.
40. Socialstyrelsen. Nationella riktlinjer för sjukdomsförebyggande metoder 2011. Tobaksbruk, riskbruk av alkohol, otillräcklig fysisk aktivitet och ohälsosamma matvanor. Stöd för styrning och ledning. Stockholm: Socialstyrelsen; 2011.
41. Smets EMA, Garssen B, Bonke B, De Haes JCJM. The multidimensional Fatigue Inventory (MFI) psychometric qualities of an instrument to assess fatigue. J Psychosom Res. 1995;39(3):315-25.
42. Wechsler D. Wechsler Adult Intelligence Scale - Third edition. San Antonio: Pearson; 1997.
43. Wechsler D. WAIS-IV technical and interpretive manual. San Antonio, Tex.: Psychological Corporation: Pearson; 2008.
44. Lezak MD, Howieson DB, Bigler ED, Tranel D. Neuropsychological Assessment. 5th ed. New York: Oxford University Press 2012.
45. Wechsler D. Wechsler Adult Intelligence Scale-Fourth Edition: Administration and scoring manual. San Antonio, TX: Psychological Corporation; 2008.
46. Delis DC, Kaplan E, Kramer JH. D-KEFS. Delis-Kaplan Executive Function System. San Antonio, TX: The Psychological Corporation; 2001.
47. Ruff RM, Allen CC. Ruff 2 & 7 Selective Attention Test. Lutz: Psychological Assessment Resources, Inc.; 1996.
48. Gunnarsson H, Grahn B, Agerstrom J. Impaired psychomotor ability and attention in patients with persistent pain: a cross-sectional comparative study. J Pain Res. 2016;9:825-35.
49. Lim J, Wen-chau W, Jiongjiong W, Detre JA, Dinges DF, Rao H. Imaging brain fatigue from sustained mental workload: An AS perfusion study of the time-on-task effect. Neuroimage. 2010;49:3426-35.
50. Treleaven J, Takasaki H. Characteristics of visual disturbances reported by subjects with neck pain. Man Ther. 2014;19(3):203-7.
51. Möller M. Fatigue and cognition - hormonal perspectives. Stockholm: Karolinska Institutet; 2013.
52. Wheelock Å M, Wheelock CE. Trials and tribulations of 'omics data analysis: assessing quality of SIMCA-based multivariate models using examples from pulmonary medicine. Mol Biosyst. 2013;9(11):2589-96.

For peer review only - http://bmjopen.bmj.com/site/about/guidelines.xhtml

STROBE Statement—Checklist of items that should be included in reports of ***cross-sectional studies***

	Item No	Recommendation	Page No
**Title and abstract**	1	(*a*) Indicate the study's design with a commonly used term in the title or the abstract	1
		(*b*) Provide in the abstract an informative and balanced summary of what was done and what was found	1
**Introduction**			
Background/rationale	2	Explain the scientific background and rationale for the investigation being reported	4-6
Objectives	3	State specific objectives, including any prespecified hypotheses	6
**Methods**			
Study design	4	Present key elements of study design early in the paper	6
Setting	5	Describe the setting, locations, and relevant dates, including periods of recruitment, exposure, follow-up, and data collection	7
Participants	6	(*a*) Give the eligibility criteria, and the sources and methods of selection of participants	7
Variables	7	Clearly define all outcomes, exposures, predictors, potential confounders, and effect modifiers. Give diagnostic criteria, if applicable	8-10
Data sources/ measurement	8*	For each variable of interest, give sources of data and details of methods of assessment (measurement). Describe comparability of assessment methods if there is more than one group	8-10
Bias	9	Describe any efforts to address potential sources of bias	11
Study size	10	Explain how the study size was arrived at	13
Quantitative variables	11	Explain how quantitative variables were handled in the analyses. If applicable, describe which groupings were chosen and why	12
Statistical methods	12	(*a*) Describe all statistical methods, including those used to control for confounding	13
		(*b*) Describe any methods used to examine subgroups and interactions	13
		(*c*) Explain how missing data were addressed	13
		(*d*) If applicable, describe analytical methods taking account of sampling strategy	N/A
		(*e*) Describe any sensitivity analyses	12
**Results**			
Participants	13*	(a) Report numbers of individuals at each stage of study—eg numbers potentially eligible, examined for eligibility, confirmed eligible, included in the study, completing follow-up, and analysed	7
		(b) Give reasons for non-participation at each stage	8
		(c) Consider use of a flow diagram	N/A
Descriptive data	14*	(a) Give characteristics of study participants (eg demographic, clinical, social) and information on exposures and potential confounders	7-8
		(b) Indicate number of participants with missing data for each variable of interest	N/A
Outcome data	15*	Report numbers of outcome events or summary measures	N/A
Main results	16	(*a*) Give unadjusted estimates and, if applicable, confounder-adjusted estimates and their precision (eg, 95% confidence interval). Make clear which confounders were adjusted for and why they were included	N/A

For peer review only - http://bmjopen.bmj.com/site/about/guidelines.xhtml

		(*b*) Report category boundaries when continuous variables were categorized	N/A
		(*c*) If relevant, consider translating estimates of relative risk into absolute risk for a meaningful time period	N/A
Other analyses	17	Report other analyses done—eg analyses of subgroups and interactions, and sensitivity analyses	N/A
**Discussion**			
Key results	18	Summarise key results with reference to study objectives	N/A
Limitations	19	Discuss limitations of the study, taking into account sources of potential bias or imprecision. Discuss both direction and magnitude of any potential bias	N/A
Interpretation	20	Give a cautious overall interpretation of results considering objectives, limitations, multiplicity of analyses, results from similar studies, and other relevant evidence	N/A
Generalisability	21	Discuss the generalisability (external validity) of the study results	N/A
**Other information**			
Funding	22	Give the source of funding and the role of the funders for the present study and, if applicable, for the original study on which the present article is based	14

*Give information separately for exposed and unexposed groups.

Note: An Explanation and Elaboration article discusses each checklist item and gives methodological background and published examples of transparent reporting. The STROBE checklist is best used in conjunction with this article (freely available on the Web sites of PLoS Medicine at http://www.plosmedicine.org/, Annals of Internal Medicine at http://www.annals.org/, and Epidemiology at http://www.epidem.com/). Information on the STROBE Initiative is available at www.strobe-statement.org.

For peer review only

For peer review only - http://bmjopen.bmj.com/site/about/guidelines.xhtml

BMJ Open

Cognitive and mental fatigue in chronic pain - cognitive functions, emotional aspects, biomarkers and neuronal correlates protocol for a descriptive cross-sectional study

Journal:	*BMJ Open*
Manuscript ID	bmjopen-2022-068011.R1
Article Type:	Protocol
Date Submitted by the Author:	21-Jan-2023
Complete List of Authors:	Möller, Marika; Karolinska Institutet, Clinical Sciences; Danderyd University Hospital,<br>Berginstrom, Nils; Umea Universitet, Psychology; Umea Universitet, Community Medicine and Rehabilitation, Rehabilitation Medicine<br>Ghafouri, B; Linköping University,<br>Holmqvist, Anna; Karolinska Institutet, Clinical Sciences; Danderyd University Hospital,<br>Lofgren, Monika; Karolinska Institutet; Danderyd University Hospital,<br>Nordin, Love; Karolinska Institutet, Department of Neurobiology, Caring Sciences and Society<br>Stålnacke, Britt-Marie; Umea Universitet, Department of Community Medicine and Rehabilitation, Rehabilitation Medicine
**Primary Subject Heading**:	Rehabilitation medicine
Secondary Subject Heading:	Neurology
Keywords:	PAIN MANAGEMENT, RADIOLOGY & IMAGING, NEUROLOGY

SCHOLARONE™
Manuscripts

Cognitive and Mental Fatigue in Chronic Pain - Cognitive Functions, Emotional Aspects, Biomarkers and Neuronal Correlates protocol for a Descriptive Cross-sectional Study.

*Möller, Marika C. [1,2], Berginström, Nils[3,4], Ghafouri, Bijar[5], Holmqvist, Anna[1,2], Löfgren, Monika[1,2], Nordin, Love[6], Stålnacke, Britt-Marie[3]

1. Department of Clinical Sciences, Karolinska Institutet, Stockholm, Sweden

2. Department of Rehabilitation Medicine, Stockholm, Danderyd University Hospital, Stockholm, Sweden

3. Department of Community Medicine and Rehabilitation, Rehabilitation Medicine, Umeå University, Umeå

4. Department of Psychology, Umeå University, Umeå

5. Pain and Rehabilitation Centre, and Department of Health, Medicine and Caring Sciences, Linköping University, Linköping, Sweden

6. Department of Neurobiology, Caring Sciences and Society, Karolinska Institutet, Stockholm, Sweden

* Corresponding author

Marika C. Möller (Orchid id: https://orcid.org/0000-0001-8700-5186)

Department of Rehabilitation Medicine

Danderyd University Hospital

Entrévägen 8

182 88 Stockholm

e-mail: marika.moller@ki.se

tfn: +46 8 12358555

Word count: Abstract: 291, manuscript: 3996
Data statement
Marika Möller

For peer review only - http://bmjopen.bmj.com/site/about/guidelines.xhtml

Abstract

Introduction Chronic pain (CP) is one of the most frequently presenting symptoms in health care and many patients with CP report mental fatigue and decline in cognitive functioning. However, the underlying mechanisms are still unknown.

Methods and analysis This study protocol describes a cross-sectional study aimed at investigating the presence of self-rated mental fatigue, objectively measured cognitive fatigability, and executive functions their relation to other cognitive functions, inflammatory biomarkers and brain connectivity in patients with CP in an outpatient setting. We will control for pain related factors such as pain intensity and secondary factors such as sleep disturbances and psychological well-being. Two hundred patients between 18 - 50 years with CP will be recruited for a neuropsychological investigation. The patients are compared with 36 healthy controls. Of these, 36 patients and 36 controls will undergo blood sampling for inflammatory markers and of these, 24 female patients and 22 female controls, between 18 – 45 years, will also undergo an fMRI investigation. Primary outcomes are cognitive fatigability, executive inhibition, imaging, and inflammatory markers. Secondary outcomes include self-rated fatigue, verbal fluency, and working memory. The study provides an approach to study fatigue and cognitive functions in CP with objective measurements and may demonstrate new models of fatigue and cognition in CP.

Ethics and dissemination The study has been approved by the Swedish Ethics Review Board (Dnr 2018/424-31; 2018/1235-32; 2018/2395-32; 2019-66148; 2022-02838-02). All patients gave their written informed concent to participate in the study. The study findings will be disseminated through publications in journals within the fields of pain, neuropsychology, and rehabilitation. Results will be spread at relevant national and international conferences, meetings, and expert forums. The results will be shared with user organizations and its members as well as relevant policy makers.

Study registration number ClinicalTrials.gov, NCT05452915. Release date: August 29, 2022

Keywords: Pain, fatigue, cognition, imaging, inflammatory markers, executive functions, attention, processing speed

For peer review only

For peer review only - http://bmjopen.bmj.com/site/about/guidelines.xhtml

ARTICLE SUMMARY

STRENGTHS AND LIMITATIONS OF THIS STUDY

- A large cross-sectional multicenter study including 200 patients and 36 controls.
- Studying fatigue in chronic pain with objective neuropsychological measurements as well as biomarkers and brain imaging is an innovative approach to find underlying pathophysiological mechanisms in this condition.
- The patients are consecutive clinical patients currently being evaluated for pain rehabilitation, which means that the results from the study are clinically generalizable.
- ~~In a longer perspective the study has potential to give rise to adapted or new rehabilitation methods for this patient group.~~
- A study limitation is that the size of the control group is matched merely for fMRI and blood sampling.

For peer review only

For peer review only - http://bmjopen.bmj.com/site/about/guidelines.xhtml

Introduction

Pain is one of the most frequently presenting symptoms in health care. When pain has been present 3 months it's classified as chronic. In Chronic Pain (CP) the central nervous system (CNS) is affected by an imbalance in the ascendent and descendent tracts. This facilitates the pain and impedes the pain inhibition. This, in turn, leads to plastic changes in the CNS, which are shown as increased sensitivity to pain and other stimuli, depression and fatigue (1-3).

Fatigue is a common complaint across CP conditions, not least in widespread pain (4-9), and it is often reported being one of the most burdensome symptoms (10). Fatigue, however is difficult to investigate because it is subjective in nature and the definition of the concept is indistinct (11). Fatigue is usually measured by questionnaires, but when using questionnaires, the results often interfere with other psychological conditions, e.g. depression (12), sleep disturbances (13) or pain (14). As depression and sleep disorders are common in patients with CP it is difficult to determine whether the fatigue seen in these patients is primarily related to pain or whether it is secondary to depression (15), which incite a general attitude that fatigue among patients with CP is due to sleep disorders and depression (16). Still, fatigue can, but need not, be linked to impaired cognitive functioning (17). Cognitive aspects of fatigue, cognitive fatigue, is a state when a cognitive demanding task give rise to a mental/subjective experience of fatigue and has often been used synonymously with mental fatigue (18). However, it is still a subjective experience. Some cognitive aspects of fatigue can also be measured on a functional level as cognitive fatigability, defined as performance decline on attention-demanding tasks (19-21).

It is well established from studies with self-reported data and cognitive tests that patients with CP are severely bothered by cognitive impairment (22-24), not least patients with generalized pain (25). Also, there are several studies showing an association between pain and deficits in attention, memory, processing speed and executive function (22, 26). It is important to separate self-reported symptoms from functional disabilities on objective tests since, as noted above, self-reports can be influenced by psychological well-being (27).

Impaired cognitive ability in CP has been explained either by "Limited resources" i.e., that the pain competes for limited attentional resources in the brain (25, 28) or by neuroplastic changes, where impaired cognitive ability in patients with CP is supposed to be caused by neurodegenerative processes. In CP the central nervous system is affected by an imbalance in the brain's pathway system, which leads to plastic changes in the brain (29). In particular, pain activates areas of the brain

For peer review only - http://bmjopen.bmj.com/site/about/guidelines.xhtml

that are important for attentional functions, which may explain why many patients with CP experience attention deficits (22).

In patients with acquired brain injuries and other neurological conditions, self-rated fatigue has been associated with impaired attention (30) and slower processing speed (31). Fatigability in cognitively demanding tasks has been shown in patients with mild traumatic brain injury (32), multiple scleroses (33) and hormonal conditions (34, 35), thus constituting an objective measure of cognitive fatigue. In acquired brain injuries and other neurological conditions cognitive fatigue has been associated to dysfunctions in the cortico-striatal networks in the basal ganglia (36-39). The relation between fatigue and attention deficits in CP has not yet been pinpointed in the literature, and cognitive fatigability has, to our knowledge, not been studied in patients with CP. It cannot be ruled out that fatigue in CP and in other neurological conditions might share underlying mechanisms, and that objectively measured cognitive fatigability could be an objective marker of cognitive fatigue also in CP.

As fatigue is an indistinctive complaint with multifactorial origin the importance of standardized taxonomy has been emphasized to clarify and improve the assessment and reporting of fatigue (11). Correlations between depression and self-rated fatigue are seen in several studies regardless of the underlying medical condition (12, 40, 41). Finding methods to capture fatigue without the result being affected by depression is important in order to disentangle other possible underlying causes to fatigue and thereby enable effective treatment. In previous studies on patients with brain injuries, cognitive fatigability has not been affected by depression (42, 43). By combining self-rating measures and fatigue measures not related to depression but rather with functional networks in the brain (32) or with results on cognitive tests (42), a clearer picture of possible underlying causes of fatigue can be obtained and in the long run also what treatment should be recommended.

Functional MRI (fMRI) may reflect increased or decreased neuronal activity in the brain as changes in neuronal activity are linked to changes in regional blood flow (rCBF) and blood oxygenation levels (BOLD). With different fMRI techniques, it is possible to study whether discrete cerebral dysfunctions can be linked to self-perceived fatigue and cognitive fatigue/fatigability on group level. Fatigue among patients with brain injuries has been linked to specific networks for attention in the brain, involving fronto-subcortical regions (44-46). Möller and Nordin have shown that fatigue in mild traumatic brain injury is related to altered connectivity in the brain (46) and that there are different neural networks that are related to subjective compared to objective measures of (32). Whether similar patterns can also be seen in patients with pain and fatigue needs to be investigated.

For peer review only - http://bmjopen.bmj.com/site/about/guidelines.xhtml

Connectivity changes have also been seen in CP, and patients with different pain conditions show connectivity changes (47). These studies have not included fatigue and fatigability and not used task-fMRI in the scanner.

Since chronic low-grade inflammation is thought to play a major role in the onset of chronic pain and some previous studies have shown elevated levels of inflammatory biomarkers in patients with chronic generalized pain and neuropathic pain (48-51), the immunological biomarkers in the present study are being investigated to improved knowledge in underlying mechanism which may lead to future potential probable diagnostic markers. A low-grade systemic inflammation may be related to fatigue and cognition as previous studies have suggested an upregulation or dysregulation of components in the immune system in patients with chronic fatigue syndrome (52, 53) and Wåhlén et al have reported significant correlation between immunity proteins and psychological distress in patients with fibromyalgia (54). As an answer to the activation of the immune system initiate activation in central immunocompetent cells such as microglia and astrocytes, resulting in a production of pro-inflammatory cytokines in the CNS that promote a change in behavior response characterized by decreased mood, increased anxiety and pain sensitivity etc. Previous studies have investigated single inflammatory markers and reported inconsistent results.

There is thus a need for increased knowledge about the association between CP, cognition and fatigue and possible underlying pathophysiological mechanisms. To improve diagnostic and prognostic models we aim to investigate the co expression profile of inflammatory biomarkers and by applying network analysis we aim to discover cellular pathways that might be activated in cognitive fatigue.

Objectives

The primary aim of the study is to investigate the presence of self-rated mental fatigue, objectively measured cognitive fatigability and executive functions and how these are related to other cognitive functions, pain related factors, inflammatory biomarkers, and brain connectivity in patients with CP.

1. Do patients with CP show more pronounced self-rated mental fatigue and cognitive fatigue compared to healthy controls?
2. Are the different fatigue measures related to cognitive functions such as process speed and attention functions?

For peer review only - http://bmjopen.bmj.com/site/about/guidelines.xhtml

3. What is the relationship between a) cognitive impairments as measured by several different cognitive tests, b) mental fatigue as measured by Multidimensional fatigue inventory-20 and VAS-f or c) cognitive fatigability as measured by WAIS-III Coding and
    a. the duration of pain
    b. generalization or intensity of pain

   and are they affected by covariates, such as sleep disorders and degree of depression/anxiety?
4. Is there an association between immunological biomarkers and a) cognitive functions, b) mental fatigue and c) cognitive fatigability?
5. Does the connectivity in the brain of female patients with CP differ from healthy controls at rest as well as during activity while performing a vigilance task?
6. Is there an association between connectivity in the brain and
    a. immunological biomarkers
    b. fatigability and
    c. results on neuropsychological tests?

For peer review only

Methods and Analysis

Study Design

This is a cross-sectional explorative case-control study. STROBE-reporting guidelines will be used (55).

Study Setting

The Unit of Pain Rehabilitation at Department of Rehabilitation Medicine at Danderyd University Hospital, Stockholm and the Department of Pain Rehabilitation, Pain Center, Umeå University Hospital, Umeå during September 2018 – October 2022. All data has been collected but no analyses have yet been published.

Patient and Public Involvement

Patients and/or the public were not involved in the design, or conduct, or reporting, or dissemination plans of this research.

Participants

Patients for Neuropsychological Assessment

Two hundred outpatients between 18 - 50 years with chronic pain (CP) from the Unit of Pain Rehabilitation at the Department of Rehabilitation Medicine at Danderyd University Hospital or from the Department of Pain Rehabilitation, Pain Center at Umeå University Hospital will be included. No

For peer review only - http://bmjopen.bmj.com/site/about/guidelines.xhtml

stratification of age will occur, but the majority of the patients will be women as they are overrepresented among those receiving rehabilitation for CP. In the analyses, the patients are compared with 36 healthy controls.

Please see flow-chart for overview of the study.

Figure 1. Flow-chart of the study

Healthy controls

Thirty-six healthy controls will be primarily used for comparison of the results from the fMRI examination and the blood sampling. They will be matched on group level in terms of gender, age, and educational level. They will be recruited among hospital staff or their friends. For those who only do neuropsychological assessment and blood sampling a token of SEK 300 will be offered and for those who do the fMRI and blood sampling a token of SEK 1000 will be offered.

Participants for fMRI

Twenty-four (24) patients, women only, between 18 – 45 years with CP and who have undergone a neuropsychological assessment at the Department of Pain Rehabilitation, Pain Center at Umeå University Hospital will also undergo an fMRI investigation. All patients fulfilling the criteria for participation in the fMRI study from Umeå the study site, are offered to participate in fMRI scanning and blood sampling, until the participation rate goal (24) is reached. They will be compared with 22 female controls from the healthy control group.

Participants for blood sampling

Of the included participants 36 patients and 36 controls will undergo blood sampling for immunological markers. All participants undergoing fMRI will also be eligible for blood sampling, as stated above. In addition, 14 patients from the Danderyd site will be consecutively recruited to the blood-sampling part of the study.

Eligibility criteria

Inclusion criteria

- disabling musculoskeletal pain for >3 months
- aged 18 - 50 years for neuropsychological assessment and 18-45 for fMRI examination

For peer review only

For peer review only - http://bmjopen.bmj.com/site/about/guidelines.xhtml

- referred to the Department of Rehabilitation Medicine at Danderyd University Hospital or to the Department of Pain Rehabilitation, Pain Center, Umeå University Hospital

Exclusion criteria

- traumatic brain injury (including concussion)
- extensive psychiatric problems or substance abuse
- previously congenital or acquired brain injury and / or does not live in their own home and who needs support in everyday life
- lack of knowledge of the Swedish language
- progressive disease
- drugs with a strong sedative effect
- pregnancy
- for fMRI examination, patients with metal objects implanted in the body or metal chips in body parts (eg after eye damage) are excluded. Patients are excluded even if they are afraid of confined spaces and if they are left-handed.

Measurements

Background information from the Swedish Quality Register for Pain Rehabilitation (SQRP) (http://www.ucr.uu.se/nrs/). Information about medication, possible trauma and background information and degree of sick leave will be obtained from the medical record.

Questionnaires from SQRP

- EuroQoL five dimensions questionnaire (EQ5D) (56)
- Hospital Anxiety and Depression Scale (HADS) (57)
- Insomnia Severity Index (ISI) (58)
- Multidimensional Pain Inventory (MPI) (59).
- Screening on physical activity by the National Board of Health and Welfare (60)

Other questionnaires

- Visual analogue scale on fatigue (VAS-f) before and after neuropsychological assessment (32)
- Visual analogue scale on fatigue (VAS-f) and pain before and after the fMRI scanning.
- Multidimensional Fatigue Inventory-20 (MFI-20) (61)
- Pain level, experienced during the neuropsychological examination, will be rated (0-10) at the end of the session.

Neuropsychological tests

For peer review only

For peer review only - http://bmjopen.bmj.com/site/about/guidelines.xhtml

- Wechsler Adult Intelligence Scale-III (WAIS-III) Digit-Symbol-Coding (Coding) measures psychomotor speed, incidental memory. The higher values the better results (62). The Coding subtest from WAIS-III will be used instead of WAIS-IV as the former has been validated for measuring fatigability (34, 43)
- WAIS-IV Matrix reasoning measures logical reasoning and premorbid cognitive level. The higher values the better results. (63)
- WAIS-IV Digit Span measures verbal attention span and working memory. The higher values the better results. (64, 65).
- Delis-Kaplan Executive Function Scale (D-KEFS) Color-Word Test and Word Fluency Test measures different aspects of executive functioning. For Color-Word test the lower values the better results and for the Word Fluency Test the higher values the better results (66).
- Ruff 2 & 7 measures automatic visual scanning and complex selective attention and processing speed). The higher values the better results (67).
- MapCog Spectra is a newly developed attention test that is administered via an iPad. The MapCog test records and analyzes the occurrence and frequency of attentional lapses and compares these to standardized norms. The standardization is based on a healthy reference group, n=313, aged 8 - 87 years. The test has a sensitivity of 95.8% and a specificity of 96% for identifying individuals with attention deficit disorder. The test is easy to perform (5-10 minutes), the data processing is automatic, the results are objective and there are no learning effects. The lower values the better results (68). We also measure fatigability by comparing the results in the end of the test with the beginning of the test.

For all test mainly raw score scores will be used in the analysis.

PRIMARY OUTCOME VARIABLES

Outcome variables are summarized in table 1.

Table 1: Outcome measures

**Primary outcome measures**	**Description**
**1. Fatigability from WAIS-III Coding**	The subject must fill in the blank spaces with the symbol which is paired to the number during 120 seconds. Cognitive fatigue is assessed by subtracting the number of digits produced in the first 30 seconds from the number of digits produced in the last 30 seconds during the full 120-second period. A non-ascending score (< 0) is considered an indicator of cognitive fatigue. Both the total value in the difference between the production between 0-30 seconds and 91-120 seconds are measured, and a dichotomized variable (non-ascending value) will be used.
**2. D-KEFS Color-Word Test**	Inhibition of over-learned verbal responses. The test has four conditions: 1) naming colors (red, blue or green, 2) reading color words printed in black,

For peer review only

For peer review only - http://bmjopen.bmj.com/site/about/guidelines.xhtml

	3) naming the color of the color words red, blue or green write in a different color than what is written, which means inhibition of an over-learned function of reading the word; 4) repeatedly switching between naming colors and reading out the printed words as quickly as possible, while at the same time the person needs to keep track of clues that indicate rule change. Contrast scores are used to examine the performance of themore complex tasks 3 and 4 and the basic tasks 1 and 2. The faster the time, the better.
**3. Fatigability on e-prime vigilance task in the fMRI scanner**	The participants are instructed to push a button as quick as they can when a set of four zeroes appears in a red rectangle and do nothing if other numbers appeared. After each response visual feedback of the reaction time is displayed. If the participant reacts at a false stimulus or if the response time of more than 1 sec. the feedback "false answer" or "no answer" is displayed respectively. The stimuli are presented at random intervals. The results are divided into quintiles and the mean reaction time is calculated for each quintile.
**4. Task-fMRI**	BOLD signal changes during the e-prime vigilance task.
**5. Resting state fMRI**	Changes in functional connectivity after performance of the e-prime vigilance task.
**6. Inflammatory markers**	Omics analyses will be used in this study. Omics studies are mainly exploratory and hypothesis generating, i.e., which proteins, metabolites and lipoproteins are identified cannot be determined in advance. Exploratory targeted analyses using panels of cytokines & chemokines and neuroinflammation will be used in combination with omics.
**Secondary outcome measures**	**Description**
**1. MFI-20**	The MFI-20 consists of five scales, based on different modes of expressing fatigue. Each scale contains four items for which the person has to indicate on a seven-point scale to what extent the particular statement applies best. 'General fatigue' includes general statements concerning a person's functioning. 'Physical fatigue' refers to the physical sensation related to the feeling of tiredness. 'Reduced activities' measures reduction in activities and 'Reduced motivation' lack of motivation. 'Mental fatigue' measures cognitive symptoms related to fatigue. Some sentences are inverted and need to be rescored. On each scales the higher values the higher fatigue.
**2. Visual Analog Scale of Fatigue**	Measurement of self-rated current fatigue level. Ranges from 0 (corresponding to no fatigue) to 10 (corresponding to the worst possible fatigue).
**3. D-KEFS Word Fluency Test**	The test measures expressive language skills, initiative and working memory and consists of three different conditions: verbal phonological flow (1) where the test person for 60 seconds produces as many unique words as possible that begins with a given letter., category flow (2) where the test person for 60 seconds per category produce as many unique words as possible in two given semantic categories and category change (3) where the test person in 60<br>seconds produce so many unique words and switch between two specified semantic categories every other time. The more words, the better the performance.

For peer review only

**4. WAIS-IV Digit Span forward and backward repetition (Attention span and working memory)**	The test person must repeat numbers that the test leader reads out the leader reads out. The number of digits is increased by one unit every two times). The test person repeats the numbers in the same order (forward repetition) or reverse order (backward repetition). Forward repetition measures auditory attention span and short-term memory, while backward repetition measures auditory working memory. Both the total number of digits and the difference between forward and backward repetition are measured.

Abbreviations: BOLD - Blood Oxygen Level Dependent; D-KEFS - Delis-Kaplan Executive Function Scale; Coding – Digit-Symbol Coding Test; fMRI - Functional magnetic resonance imaging; MFI - Multidimensional Fatigue Inventory; WAIS– Wechsler Adult Intelligence Scale

Data Collection and Management

Recruitment

The pain rehabilitation unit at the Department of Rehabilitation Medicine, Danderyd University Hospital and the Department of Pain Rehabilitation, Pain Center at Umeå University Hospital offer team assessment and evidence-based team-based multimodal rehabilitation for patients with complex CP (>3 months). To receive rehabilitation at the clinics, a referral including a pain assessment is required. Information about the study is sent to the referred patients at the same time as the call for initial assessment.

At time for the team assessment, the patient is asked orally by the team nurse if the testing psychologist may contact the patient to inform about the study. The patient's consent is logged, and a log is made of the number who say yes and the number and gender and age of those who say no. No personal data other than gender will be registered on those who say no. The purpose of the log is only to be able to assess generalizability based on the proportion who have agreed to participate in the study.

The testing psychologist then calls the patients and inform them about the study and if the patient agrees, an appointment is made for neuropsychological. In connection with the assessment, the psychologist ensures that they have understood the information about the study and that participation is voluntary, inquire about whether they have further questions and collect the signed consent form. Female patients who are investigated in Umeå are asked if they want to participate in the fMRI study. The patients are assessed by an independent psychologist, who does not treat the patients clinically.

The study patients will be compared with healthy controls. Healthy controls will be recruited through advertising among hospital staff. Matching at group level will take place regarding age, gender and level of education.

Imaging

For peer review only

For peer review only - http://bmjopen.bmj.com/site/about/guidelines.xhtml

In a 3-tesla MR scanner, conventional anatomical MRI sequences will be analyzed as well as sequences for detailed anatomical assessment. We use an established measurement paradigm that has previously been used on healthy subjects (69) and patients with mild traumatic brain injury (70).

All participants are examined with fMRI during a 20-minute vigilance (reaction time) task (e-prime), where fatigue is measured by recording mean reaction time / quintile. BOLD resting-state fMRI examination is performed before and after the task. In addition, MRI protocol will include standard clinical protocols that include high-resolution T1-weighted sequences and high-resolution T2-weighted Scan (FLAIR). All structural images are reviewed by a radiologist to scan for incidental findings within the brain.

The investigation is completely non-invasive and takes about 60 minutes.

Blood sampling

Venous blood samples (10-20 ml) are collected in P100 tubes from BD diagnostic, containing a protease inhibitor cocktail that prevents protein cleavage / degradation) from each subject. The blood samples are centrifuged to remove red blood cells. The plasma fraction is transferred to a new tube, portioned and stored at -86 ° C until analysis at PAINOMICS lab in Linköping, Sweden.

Plasma samples will be analyzed using antibody-based methods such as multiplex technology (Meso Scale Diagnostics, Rockville, MD). Instrument, MSD Technology platform, which is a multiplex instrument, which can analyze up to 92 different substances (cytokines, chemokines, growth factors and metabolic proteins) in one go. Omics analysis consisted of proteomics, metabolomics and lipoprotein profiling will be used to identify other inflammatory markers than those analyzed by the antibody-based (targeted analysis) method.

Data Management

All data material will be recorded with a participant ID and will be unidentifiable for those who do not have access to the code-key. In the statistical processing, the participants will be coded. Code key is stored separately from data and only pseudonymized data will be shared between the two sites. De-identified data will be electronically stored on the server at the two sites and will be deleted 10 years after the project has ended. The final dataset will be available to researchers actively contributing to statistical analyses and publications. Data entry will be controlled by initial exploratory analyses, including range checks, in order to promote data quality. Regarding biological specimens the samples of blood will be centrifuged and plasma will be aliquoted and stored at the University Hospital, with special notification to the biobank. The samples will be blinded before analysis. This study has a hypothesis generating character as we use proteomic methods therefore

For peer review only

samples (that are not thawed) will be stored for future analysis according to approved ethical application.

Sample Size

Power calculation on the primary fatigue measure the WAIS-III Coding Test from WAIS-III on: For a mean difference between patients and controls of 2.5 and standard deviation of 3.0 and alpha level of 5% (two-sided) and strength of 80%, a group of 24 participants in each group is a sufficiently large sample (71). This size also holds for the fMRI sub study as an earlier study on mild traumatic brain injury has shown that 10 patients in each group was sufficient (46). The biomarkers data will be analyzed using multivariate data analysis (MVDA). Algorithms for sample size calculations using MVDA do not exist. MVDA is designed to handle a few subjects, low to variable ratios, and multiple intercorrelated variables. Based on our previous biomarkers study (49, 72), the sample size in this study will be sufficient to detect a difference between groups”**.**

Confidentialty

Information on participants will be handled by health care professionals adhering to Swedish Law ensuring confidentiality and data protection by coding of individual participants’ collected data. Results and data will be presented at a group level in publications, rendering identification of individual patients impossible. All data will be stored accordance with the General Data Protection Regulation (GDPR). The data for participants from Stockholm area will be stored at a password protected project server in Danderyd University Hospital and for patients from Umeå region at Umeå University. Data will not be accessed by unauthorized persons.

Data analysis

For all study questions descriptive statistics will be used to depict demographics, injury characteristics, results on neuropsychological tests and psychological screening instruments. Multiple linear and logistic regression will be used to detect any interaction effects. Parametric methods such as independent t-test, One-sample t-test (comparing with standardized test norms), Pearson correlation, multiple linear regression) will be used when data is normally distributed and non-parametric methods (Man-Whitney U-test, Spearman correlation, Chi2, logistic regression) when data is skewed or on interval level or lower. Missing data will be listed as data loss. The significance level will be set to <.05 (two-tailed).

For peer review only

Concerning study question 4 data from omics and targeted analysis will be analyzed using advanced multivariate data analysis using SIMCA-P+, that is recommended method in omics where it account for multicollinearity problems and missing data and when the numbers of variable exceed the number of observations (73). MVDA will be used for analysis of the biomarkers. Principal component analysis (PCA), Orthogonal partial least squares- discriminant analysis (OPLS-DA), and OPLS regression will be applied for analysis of the biomarkers and their ability to differentiate between groups and their correlation with clinical characteristics and outcomes. PCA is an unsupervised analysis that reduces the dimensionality of data and extract and displays systematic variation in the data matrix. Outliers will be identified using score plots in combination with Hotelling's T2 test (the distance from the origin in the model plane for each selected observation) and DModX (the distance of the observation to the X model plane). The values that are larger than the 95% confidence limit are considered as suspect and the values larger than the 99% limit should be considered as strong outliers (74).

OPLS-DA will be used to identify biomarkers responsible for group separation. The OPLS-DA model reveals variables as loadings and the higher the value of a loading the more important it is for the model, e.g., group separation. This can be measured as Variable influence on projection (VIP) values. The p(corr) is the loading of each variable scaled as a correlation coefficient (ranging from -1 to +1). An absolute p(corr) of >0.4-0.5 and VIP > 1.0 are considered as significant (73). Other model diagnosis parameters that are considered are R2 value- which describes the "goodness of fit", i.e., the fraction of sum of squares of all the variables explained by a principal component. The Q2 value- describes the "goodness of prediction", i.e., the fraction of the total variation of the variables that can be predicted by a principal component using a cross validation method (74) and cross-validated analysis of variance (CV-ANOVA). The R2 must not differ more than 0.2-0.3 compared to Q2 and CV-ANOVA< 0.05 is considered as a significant model.

Regarding study question 5 analysis of fMRI data will be done with special software for MRI analysis, developed at Oxford University (http://www.fmrib.ox.ac.uk/fsl/). The resting state data will be analyzed using FSL tools such as MELODIC in order to identify spatial and temporal components by independent component analysis at group level. All relevant preprocessing steps will be performed, such as motion correction, registration to a standard template and filtering of low frequency drifts. Group differences between patients and controls will be analyzed using FSL dual regression. The fMRI data will be analyzed using both MELODIC and FEAT which is an FSL tool based on general linear modelling performing multiple regression on the fMRI data and the functional paradigm.

For study question 6 multiple linear and logistic regression will be used to detect any interaction effects.

For peer review only - http://bmjopen.bmj.com/site/about/guidelines.xhtml

PLANS FOR COMMUNICATING IMPORTANT PROTOCOL AMENDMENTS TO RELEVANT PARTIES

Important protocol modifications will be reported to the Ethics committee in Sweden and amendments will be made to the trial registry (Clinicaltrials.gov).

PATIENT AND PUBLIC INVOLVEMENT

Patients were not involved in formulating the research question or setting the research design for the planned study.

ETHICS AND DISSEMINATION

The study was approved by the Ethics Review Board (2018/424-31; 2018/1235-32; 2018/2395-32; 2019-66148; 2022-02838-02). All participants are provided oral and written information by the neuropsychologists (AH and NB) and will give written informed consent to participate in the study to them.

Publications are planned for journals in the fields of pain, neuropsychology, and rehabilitation. Results will further be spread at relevant conferences, national and international meetings, and expert forums. The results will be shared with user organizations and its members as well as relevant policy makers.

AUTHOR CONTRIBUTIONS

MCM, ML and BMS formed the original research concept. BG, LN, NB and AH contributed to the study design within their field of expertise. MCM and BMS will coordinate the project in Stockholm and Umeå in cooperation with NB, BG, AH, ML, and LN. NB and AH will be responsible for data collection and statistical analyses of the neuropsychological and demographic data in collaboration with MCM, ML and BMS. LN will be responsible for fMRI analyses in collaboration with NB. BG will be responsible for the analyses of biomarkers. MCM has written the manuscript draft. All authors have contributed with important intellectual content to the manuscript. Regarding upcoming manuscripts, we will use authorship eligibility guidelines. We will not use professional writers.

FUNDING

For peer review only - http://bmjopen.bmj.com/site/about/guidelines.xhtml

This study is supported by Promobilia Foundation, grant number [A22056] the research and development fund granted by the County Council of Västerbotten [RV-928951; RV-939460], and through a regional agreement between Umeå University and Västerbotten County Council (ALF) [RV-930845; RV-940388; RV-967132]. The Department of Rehabilitation Medicine and the Karolinska Institutet, Department of Clinical Sciences, Danderyd University Hospital have also provided valuable support for the study to be feasible. The funders have no vested interest in the study and have not influenced the design.

COMPETING INTERESTS

None declared.

PATIENT CONSENT FOR PUBLICATION

Not Applicable

REFERENCES

1. Ng SK, Urquhart DM, Fitzgerald PB, Cicuttini FM, Hussain SM, Fitzgibbon BM. The Relationship between Structural and Functional Brain Changes and Altered Emotion and Cognition in Chronic Low Back Pain: A Systematic Review of MRI and fMRI Studies. Clin J Pain. 2017.
2. Flodin P, Martinsen S, Mannerkorpi K, Lofgren M, Bileviciute-Ljungar I, Kosek E, et al. Normalization of aberrant resting state functional connectivity in fibromyalgia patients following a three month physical exercise therapy. Neuroimage Clin. 2015;9:134-9.
3. Marchand S. The physiology of pain mechanisms: from the periphery to the brain. Rheum Dis Clin North Am. 2008;34(2):285-309.
4. Creavin ST, Dunn KM, Mallen CD, Nijrolder I, van der Windt DA. Co-occurrence and associations of pain and fatigue in a community sample of Dutch adults. Eur J Pain. 2010;14(3):327-34.
5. Hart RP, Martelli MF, Zasler ND. Chronic pain and neuropsychological functioning. Neuropsychol Rev. 2000;10(3):131-49.
6. Wolfe F, Hawley DJ, Wilson K. The prevalence and meaning of fatigue in rheumatic disease. J Rheumatol. 1996;23(8):1407-17.
7. Bjurstrom MF, Irwin MR. Polysomnographic characteristics in nonmalignant chronic pain populations: A review of controlled studies. Sleep Med Rev. 2016;26:74-86.
8. Fishbain DA, Cole B, Cutler RB, Lewis J, Rosomoff HL, Fosomoff RS. Is pain fatiguing? A structured evidence-based review. Pain Med. 2003;4(1):51-62.
9. Louati K, Berenbaum F. Fatigue in chronic inflammation - a link to pain pathways. Arthritis Res Ther. 2015;17:254.
10. Arnold LM, Crofford LJ, Mease PJ, Burgess SM, Palmer SC, Abetz L, et al. Patient perspectives on the impact of fibromyalgia. Patient Educ Couns. 2008;73(1):114-20.
11. Kluger BM, Krupp LB, Enoka RM. Fatigue and fatigability in neurologic illnesses: proposal for a unified taxonomy. Neurology. 2013;80(4):409-16.
12. Arnold LM. Understanding fatigue in major depressive disorder and other medical disorders. Psychosomatics. 2008;49(3):185-90.
13. Akerstedt T, Knutsson A, Westerholm P, Theorell T, Alfredsson L, Kecklund G. Mental fatigue, work and sleep. J Psychosom Res. 2004;57(5):427-33.
14. Snekkevik H, Eriksen HR, Tangen T, Chalder T, Reme SE. Fatigue and depression in sick-listed chronic low back pain patients. Pain Med. 2014;15(7):1163-70.
15. Mease P, Arnold LM, Bennett R, Boonen A, Buskila D, Carville S, et al. Fibromyalgia syndrome. J Rheumatol. 2007;34(6):1415-25.

For peer review only - http://bmjopen.bmj.com/site/about/guidelines.xhtml

16. Kurtze N, Svebak S. Fatigue and patterns of pain in fibromyalgia: correlations with anxiety, depression and co-morbidity in a female county sample. Br J Med Psychol. 2001;74(Pt 4):523-37.
17. Johnson SK, Lange G, DeLuca J, Korn LR, Natelson B. The effects of fatigue on neuropsychological performance in patients with chronic fatigue syndrome, multiple sclerosis, and depression. Appl Neuropsychol. 1997;4(3):145-53.
18. Wylie GR, Flashman LA. Understanding the interplay between mild traumatic brain injury and cognitive fatigue: models and treatments. Concussion (London, England). 2017;2(4):Cnc50.
19. Schwid SR, Tyler CM, Scheid EA, Weinstein A, Goodman AD, McDermott MP. Cognitive fatigue during a test requiring sustained attention: a pilot study. Mult Scler. 2003;9(5):503-8.
20. Bryant D, Chiaravalloti ND, DeLuca J. Objective Measurement of Cognitive Fatigue in Multiple Sclerosis. Rehabilitation psychology. 2004;49(2):114.
21. Walker LAS, Lindsay-Brown AP, Berard JA. Cognitive Fatigability Interventions in Neurological Conditions: A Systematic Review. Neurology and therapy. 2019;8(2):251-71.
22. Moriarty O, McGuire BE, Finn DP. The effect of pain on cognitive function: a review of clinical and preclinical research. Prog Neurobiol. 2011;93(3):385-404.
23. Ferreira Kdos S, Oliver GZ, Thomaz DC, Teixeira CT, Foss MP. Cognitive deficits in chronic pain patients, in a brief screening test, are independent of comorbidities and medication use. Arq Neuropsiquiatr. 2016;74(5):361-6.
24. Rathbone M, Parkinson W, Rehman Y, Jiang S, Bhandari M, Kumbhare D. Magnitude and variability of effect sizes for the associations between chronic pain and cognitive test performances: a meta-analysis. Br J Pain. 2016;10(3):141-55.
25. Grisart J, Van der Linden M, Masquelier E. Controlled processes and automaticity in memory functioning in fibromyalgia patients: relation with emotional distress and hypervigilance. J Clin Exp Neuropsychol. 2002;24(8):994-1009.
26. Higgins DM, Martin AM, Baker DG, Vasterling JJ, Risbrough V. The Relationship Between Chronic Pain and Neurocognitive Function: A Systematic Review. Clin J Pain. 2018;34(3):262-75.
27. Lundin A, de Boussard C, Edman G, Borg J. Symptoms and disability until 3 months after mild TBI. Brain Inj. 2006;20(8):799-806.
28. Legrain V, Damme SV, Eccleston C, Davis KD, Seminowicz DA, Crombez G. A neurocognitive model of attention to pain: behavioral and neuroimaging evidence. Pain. 2009;144(3):230-2.
29. Ng K, Sin , Urquhart M, Donna , Fitzgerald B, Paul , Cicuttini M, Flavia , Hussain M, Sultana , Fitzgibbon M, Bernadette. The Relationship Between Structural and Functional Brain Changes and Altered Emotion and Cognition in Chronic Low Back Pain Brain Changes: A Systematic Review of MRI and fMRI Studies. The Clinical Journal of Pain. 2018;34(3):237-61.
30. Ziino C, Ponsford J. Measurement and prediction of subjective fatigue following traumatic brain injury. J Int Neuropsychol Soc. 2005;11(4):416-25.
31. Johansson B, Berglund P, Rönnbäck L. Mental fatigue and impaired information processing after mild and moderate traumatic brain injury. Brain Inj. 2009;23(13-14):1027-40.
32. Möller MC, Nordin LE, Bartfai A, Julin P, Li TQ. Fatigue and Cognitive Fatigability in Mild Traumatic Brain Injury are Correlated with Altered Neural Activity during Vigilance Test Performance. Front Neurol. 2017;8:496.
33. Walker LA, Berard JA, Berrigan LI, Rees LM, Freedman MS. Detecting cognitive fatigue in multiple sclerosis: method matters. J Neurol Sci. 2012;316(1-2):86-92.
34. Möller MC, Bartfai A, Nygren de Boussard C, Flöter Rådestad A, Calissendorff J. High rates of fatigue in newly diagnosed Graves' disease. Fatigue: Biomedicine, Health & Behavior. 2014;2(3):153-62.
35. Möller MC, Rådestad AF, von Schoultz B, Bartfai A. Effect of estrogen and testosterone replacement therapy on cognitive fatigue. Gynecol Endocrinol. 2013;29(2):173-6.
36. Chaudhuri A, Behan PO. Fatigue and basal ganglia. J Neurol Sci. 2000;179(S 1-2):34-42.
37. DeLuca J, Genova HM, Hillary FG, Wylie G. Neural correlates of cognitive fatigue in multiple sclerosis using functional MRI. J Neurol Sci. 2008;270(1-2):28-39.
38. Kohl AD, Wylie GR, Genova HM, Hillary FG, Deluca J. The neural correlates of cognitive fatigue in traumatic brain injury using functional MRI. Brain Inj. 2009;23(5):420-32.
39. Chaudhuri A, Behan PO. Fatigue in neurological disorders. Lancet. 2004;363(9413):978-88.
40. Holmqvist A, Lindstedt MB, Moller MC. Relationship between fatigue after acquired brain injury and depression, injury localization and aetiology: An explorative study in a rehabilitation setting. J Rehabil Med. 2018;50(8):725-31.

For peer review only

41. van Barneveld E, Manders J, van Osch FHM, van Poll M, Visser L, van Hanegem N, et al. Depression, Anxiety, and Correlating Factors in Endometriosis: A Systematic Review and Meta-Analysis. J Womens Health (Larchmt). 2022;31(2):219-30.
42. Holmqvist A, Bartfai A, Markovic G, Möller MC. Does Intensive Training of Attention Influence Cognitive Fatigability in Patients With Acquired Brain Injury? Front Neurosci. 2021;15:656876.
43. Möller MC, Nygren de Boussard C, Oldenburg C, Bartfai A. An investigation of attention, executive, and psychomotor aspects of cognitive fatigability. J Clin Exp Neuropsychol [Internet]. 2014; 26:[1-14 pp.].
44. Cantor F. Central and peripheral fatigue: exemplified by multiple sclerosis and myasthenia gravis. Pm & R. 2010;2(5):399-405.
45. Dobryakova E, Deluca J, Genova HM, Wylie GR. Neural Correlates of Cognitive Fatigue: Cortico-Striatal Circuitry and Effort-Reward Imbalance. Journal Of The International Neuropsychological Society: JINS. 2013:1-5.
46. Nordin LE, Moller MC, Julin P, Bartfai A, Hashim F, Li TQ. Post mTBI fatigue is associated with abnormal brain functional connectivity. Sci Rep. 2016;6:21183.
47. Apkarian AV. Frontiers in Neuroscience. Human Brain Imaging Studies of Chronic Pain: Translational Opportunities. In: Kruger L, Light AR, editors. Translational Pain Research: From Mouse to Man. Boca Raton, FL: CRC Press/Taylor & Francis Llc.; 2010.
48. Gerdle B, Backryd E, Falkenberg T, Lundstrom E, Ghafouri B. Changes in inflammatory plasma proteins from patients with chronic pain associated with treatment in an interdisciplinary multimodal rehabilitation program - an explorative multivariate pilot study. Scandinavian journal of pain. 2019.
49. Jönsson M, Gerdle B, Ghafouri B, Bäckryd E. The inflammatory profile of cerebrospinal fluid, plasma, and saliva from patients with severe neuropathic pain and healthy controls-a pilot study. BMC Neurosci. 2021;22(1):6.
50. Olausson P, Gerdle B, Ghafouri N, Sjöström D, Blixt E, Ghafouri B. Protein alterations in women with chronic widespread pain--An explorative proteomic study of the trapezius muscle. Sci Rep. 2015;5:11894.
51. Wåhlén K, Olausson P, Carlsson A, Ghafouri N, Gerdle B, Ghafouri B. Systemic alterations in plasma proteins from women with chronic widespread pain compared to healthy controls: a proteomic study. J Pain Res. 2017;10:797-809.
52. Mandarano AH, Maya J, Giloteaux L, Peterson DL, Maynard M, Gottschalk CG, et al. Myalgic encephalomyelitis/chronic fatigue syndrome patients exhibit altered T cell metabolism and cytokine associations. J Clin Invest. 2020;130(3):1491-505.
53. Lorusso L, Mikhaylova SV, Capelli E, Ferrari D, Ngonga GK, Ricevuti G. Immunological aspects of chronic fatigue syndrome. Autoimmun Rev. 2009;8(4):287-91.
54. Wåhlén K, Ernberg M, Kosek E, Mannerkorpi K, Gerdle B, Ghafouri B. Significant correlation between plasma proteome profile and pain intensity, sensitivity, and psychological distress in women with fibromyalgia. Sci Rep. 2020;10(1):12508.
55. von Elm E, Altman DG, Egger M, Pocock SJ, Gøtzsche PC, Vandenbroucke JP. The Strengthening the Reporting of Observational Studies in Epidemiology (STROBE) statement: guidelines for reporting observational studies. Ann Intern Med. 2007;147(8):573-7.
56. Group E. EuroQol--a new facility for the measurement of health-related quality of life. Health policy (Amsterdam, Netherlands). 1990;16(3):199-208.
57. Zigmond AS, Snaith RP. The Hospital Anxiety and Depression Scale. Acta Psychiatrica Scandinavica. 1983.
58. Bastien CH, Vallieres A, Morin CM. Validation of the Insomnia Severity Index as an outcome measure for insomnia research. Sleep Med. 2001;2(4):297-307.
59. Kerns RD, Turk DC, Rudy TE. The West Haven-Yale Multidimensional Pain Inventory (WHYMPI). Pain. 1985;23(4):345-56.
60. Socialstyrelsen. Nationella riktlinjer för sjukdomsförebyggande metoder 2011. Tobaksbruk, riskbruk av alkohol, otillräcklig fysisk aktivitet och ohälsosamma matvanor. Stöd för styrning och ledning. Stockholm: Socialstyrelsen; 2011.
61. Smets EMA, Garssen B, Bonke B, De Haes JCJM. The multidimensional Fatigue Inventory (MFI) psychometric qualities of an instrument to assess fatigue. J Psychosom Res. 1995;39(3):315-25.
62. Wechsler D. Wechsler Adult Intelligence Scale - Third edition. San Antonio: Pearson; 1997.
63. Wechsler D. WAIS-IV technical and interpretive manual. San Antonio, Tex.: Psychological Corporation: Pearson; 2008.
64. Lezak MD, Howieson DB, Bigler ED, Tranel D. Neuropsychological Assessment. 5th ed. New York: Oxford University Press 2012.

For peer review only - http://bmjopen.bmj.com/site/about/guidelines.xhtml

65. Wechsler D. Wechsler Adult Intelligence Scale-Fourth Edition: Administration and scoring manual. San Antonio, TX: Psychological Corporation; 2008.
66. Delis DC, Kaplan E, Kramer JH. D-KEFS. Delis-Kaplan Executive Function System. San Antonio, TX: The Psychological Corporation; 2001.
67. Ruff RM, Allen CC. Ruff 2 & 7 Selective Attention Test. Lutz: Psychological Assessment Resources, Inc.; 1996.
68. Gunnarsson H, Grahn B, Agerstrom J. Impaired psychomotor ability and attention in patients with persistent pain: a cross-sectional comparative study. J Pain Res. 2016;9:825-35.
69. Lim J, Wen-chau W, Jiongjiong W, Detre JA, Dinges DF, Rao H. Imaging brain fatigue from sustained mental workload: An AS perfusion study of the time-on-task effect. Neuroimage. 2010;49:3426-35.
70. Treleaven J, Takasaki H. Characteristics of visual disturbances reported by subjects with neck pain. Man Ther. 2014;19(3):203-7.
71. Möller M. Fatigue and cognition - hormonal perspectives. Stockholm: Karolinska Institutet; 2013.
72. Ghafouri B, Edman E, Löf M, Lund E, Leinhard OD, Lundberg P, et al. Fibromyalgia in women: association of inflammatory plasma proteins, muscle blood flow, and metabolism with body mass index and pain characteristics. Pain Rep. 2022;7(6):e1042.
73. Wheelock Å M, Wheelock CE. Trials and tribulations of 'omics data analysis: assessing quality of SIMCA-based multivariate models using examples from pulmonary medicine. Mol Biosyst. 2013;9(11):2589-96.
74. Eriksson L, Johansson E, Kettaneh-Wold N, Trygg J, Wikström C, Wold S. Multi- and Megavariate Data Analysis; Part I and II. 2 ed. Umeå Umetrics AB; 2006.

FIGURE CAPTION

Figure 1. Flow-chart of the study.

For peer review only

For peer review only - http://bmjopen.bmj.com/site/about/guidelines.xhtml

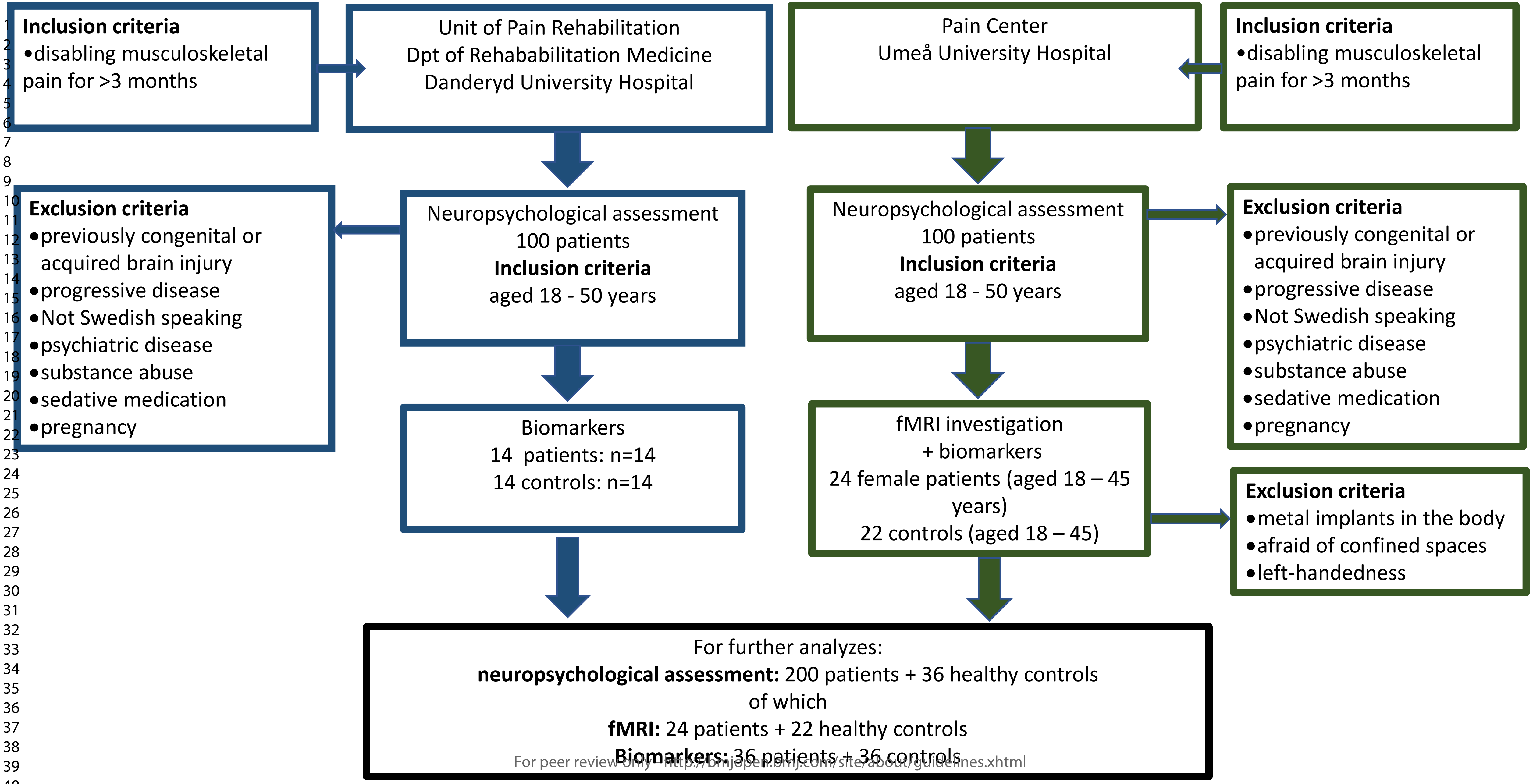

For peer review only - http://bmjopen.bmj.com/site/about/guidelines.xhtml

STROBE Statement—Checklist of items that should be included in reports of ***cross-sectional studies***

	Item No	Recommendation	Page No
**Title and abstract**	1	(*a*) Indicate the study's design with a commonly used term in the title or the abstract	1
		(*b*) Provide in the abstract an informative and balanced summary of what was done and what was found	1
**Introduction**			
Background/rationale	2	Explain the scientific background and rationale for the investigation being reported	4-6
Objectives	3	State specific objectives, including any prespecified hypotheses	6
**Methods**			
Study design	4	Present key elements of study design early in the paper	6
Setting	5	Describe the setting, locations, and relevant dates, including periods of recruitment, exposure, follow-up, and data collection	7
Participants	6	(*a*) Give the eligibility criteria, and the sources and methods of selection of participants	7
Variables	7	Clearly define all outcomes, exposures, predictors, potential confounders, and effect modifiers. Give diagnostic criteria, if applicable	8-10
Data sources/ measurement	8*	For each variable of interest, give sources of data and details of methods of assessment (measurement). Describe comparability of assessment methods if there is more than one group	8-10
Bias	9	Describe any efforts to address potential sources of bias	11
Study size	10	Explain how the study size was arrived at	13
Quantitative variables	11	Explain how quantitative variables were handled in the analyses. If applicable, describe which groupings were chosen and why	12
Statistical methods	12	(*a*) Describe all statistical methods, including those used to control for confounding	13
		(*b*) Describe any methods used to examine subgroups and interactions	13
		(*c*) Explain how missing data were addressed	13
		(*d*) If applicable, describe analytical methods taking account of sampling strategy	N/A
		(*e*) Describe any sensitivity analyses	12
**Results**			
Participants	13*	(a) Report numbers of individuals at each stage of study—eg numbers potentially eligible, examined for eligibility, confirmed eligible, included in the study, completing follow-up, and analysed	7
		(b) Give reasons for non-participation at each stage	8
		(c) Consider use of a flow diagram	N/A
Descriptive data	14*	(a) Give characteristics of study participants (eg demographic, clinical, social) and information on exposures and potential confounders	7-8
		(b) Indicate number of participants with missing data for each variable of interest	N/A
Outcome data	15*	Report numbers of outcome events or summary measures	N/A
Main results	16	(*a*) Give unadjusted estimates and, if applicable, confounder-adjusted estimates and their precision (eg, 95% confidence interval). Make clear which confounders were adjusted for and why they were included	N/A

For peer review only - http://bmjopen.bmj.com/site/about/guidelines.xhtml

		(*b*) Report category boundaries when continuous variables were categorized	N/A
		(*c*) If relevant, consider translating estimates of relative risk into absolute risk for a meaningful time period	N/A
Other analyses	17	Report other analyses done—eg analyses of subgroups and interactions, and sensitivity analyses	N/A
**Discussion**			
Key results	18	Summarise key results with reference to study objectives	N/A
Limitations	19	Discuss limitations of the study, taking into account sources of potential bias or imprecision. Discuss both direction and magnitude of any potential bias	N/A
Interpretation	20	Give a cautious overall interpretation of results considering objectives, limitations, multiplicity of analyses, results from similar studies, and other relevant evidence	N/A
Generalisability	21	Discuss the generalisability (external validity) of the study results	N/A
**Other information**			
Funding	22	Give the source of funding and the role of the funders for the present study and, if applicable, for the original study on which the present article is based	14

*Give information separately for exposed and unexposed groups.

Note: An Explanation and Elaboration article discusses each checklist item and gives methodological background and published examples of transparent reporting. The STROBE checklist is best used in conjunction with this article (freely available on the Web sites of PLoS Medicine at http://www.plosmedicine.org/, Annals of Internal Medicine at http://www.annals.org/, and Epidemiology at http://www.epidem.com/). Information on the STROBE Initiative is available at www.strobe-statement.org.

For peer review only

For peer review only - http://bmjopen.bmj.com/site/about/guidelines.xhtml

BMJ Open

Cognitive and mental fatigue in chronic pain—cognitive functions, emotional aspects, biomarkers, and neuronal correlates: protocol for a descriptive cross-sectional study

Journal:	*BMJ Open*
Manuscript ID	bmjopen-2022-068011.R2
Article Type:	Protocol
Date Submitted by the Author:	21-Mar-2023

Complete List of Authors:	Möller, Marika; Karolinska Institutet, Clinical Sciences; Danderyd University Hospital,<br>Berginstrom, Nils; Umea Universitet, Psychology; Umea Universitet, Community Medicine and Rehabilitation, Rehabilitation Medicine<br>Ghafouri, B; Linköping University,<br>Holmqvist, Anna; Karolinska Institutet, Clinical Sciences; Danderyd University Hospital,<br>Lofgren, Monika; Karolinska Institutet; Danderyd University Hospital,<br>Nordin, Love; Karolinska Institutet, Department of Neurobiology, Caring Sciences and Society<br>Stålnacke, Britt-Marie; Umea Universitet, Department of Community Medicine and Rehabilitation, Rehabilitation Medicine

**Primary Subject Heading**:	Rehabilitation medicine
Secondary Subject Heading:	Neurology
Keywords:	PAIN MANAGEMENT, RADIOLOGY & IMAGING, NEUROLOGY

SCHOLARONE™
Manuscripts

Cognitive and mental fatigue in chronic pain—cognitive functions, emotional aspects, biomarkers, and neuronal correlates: protocol for a descriptive cross-sectional study

*Möller, Marika C. [1,2], Berginström, Nils[3,4], Ghafouri, Bijar[5], Holmqvist, Anna[1,2], Löfgren, Monika[1,2], Nordin, Love[6], Stålnacke, Britt-Marie[3]

1. Department of Clinical Sciences, Karolinska Institutet, Stockholm, Sweden

2. Department of Rehabilitation Medicine, Stockholm, Danderyd University Hospital, Stockholm, Sweden

3. Department of Community Medicine and Rehabilitation, Rehabilitation Medicine, Umeå University, Umeå, Sweden

4. Department of Psychology, Umeå University, Umeå, Sweden

5. Pain and Rehabilitation Centre, and Department of Health, Medicine and Caring Sciences, Linköping University, Linköping, Sweden

6. Department of Neurobiology, Caring Sciences and Society, Karolinska Institutet, Stockholm, Sweden

*Correspondence to:

Marika C. Möller (ORCID iD: https://orcid.org/0000-0001-8700-5186)

Department of Rehabilitation Medicine, Danderyd University Hospital, Entrévägen 8, 182 88 Stockholm, Sweden

e-mail: marika.moller@ki.se

Word count: Abstract: 291, manuscript: 3996

For peer review only

Abstract

Introduction Chronic pain (CP) is one of the most frequently presenting conditions in health care and many patients with CP report mental fatigue and a decline in cognitive functioning. However, the underlying mechanisms are still unknown.

Methods and analysis This study protocol describes a cross-sectional study aimed at investigating the presence of self-rated mental fatigue, objectively measured cognitive fatigability, and executive functions and their relation to other cognitive functions, inflammatory biomarkers, and brain connectivity in patients with CP. We will control for pain-related factors such as pain intensity and secondary factors such as sleep disturbances and psychological well-being. Two hundred patients 18 - 50 years with CP will be recruited for a neuropsychological investigation at two outpatient study centers in Sweden. The patients are compared with 36 healthy controls. Of these, 36 patients and 36 controls will undergo blood sampling for inflammatory markers and of these, 24 female patients and 22 female controls, between 18 – 45 years, will undergo an fMRI investigation. Primary outcomes are cognitive fatigability, executive inhibition, imaging, and inflammatory markers. Secondary outcomes include self-rated fatigue, verbal fluency, and working memory. The study provides an approach to study fatigue and cognitive functions in CP with objective measurements and may demonstrate new models of fatigue and cognition in CP.

Ethics and dissemination The study has been approved by the Swedish Ethics Review Board (Dnr 2018/424-31; 2018/1235-32; 2018/2395-32; 2019-66148; 2022-02838-02). All patients gave written informed consent to participate in the study. The study findings will be disseminated through publications in journals within the fields of pain, neuropsychology, and rehabilitation. Results will be spread at relevant national and international conferences, meetings, and expert forums. The results will be shared with user organizations and their members as well as relevant policymakers.

Study registration ClinicalTrials.gov, NCT05452915.

Keywords: Pain, fatigue, cognition, imaging, inflammatory markers, executive functions, attention, processing speed

For peer review only

ARTICLE SUMMARY

STRENGTHS AND LIMITATIONS OF THIS STUDY

- A large cross-sectional multicenter study including 200 patients and 36 controls.
- Studying fatigue in chronic pain with objective neuropsychological measurements as well as biomarkers and brain imaging is an innovative approach to finding underlying pathophysiological mechanisms in this condition.
- The patients are consecutive clinical patients currently being evaluated for pain rehabilitation, which means that the results from the study are clinically generalizable.
- A study limitation is the small size of the control group; however, the size of the control group is adapted for comparison of the results from the fMRI examination and blood sampling.

For peer review only

For peer review only - http://bmjopen.bmj.com/site/about/guidelines.xhtml

INTRODUCTION

Pain is one of the most frequently presenting conditions in health care. When pain has been present for 3 months it's classified as chronic. In Chronic Pain (CP), the central nervous system (CNS) is affected by an imbalance in the ascendent and descendent tracts. This facilitates the pain and impedes pain inhibition. This, in turn, leads to plastic changes in the CNS, which are shown as increased sensitivity to pain and other stimuli, depression, and fatigue (1-3).

Fatigue is a common complaint across CP conditions, not least in widespread pain (4-9), and it is often reported to be one of the most burdensome symptoms (10). Fatigue, however, is difficult to investigate because it is subjective in nature and the definition of the concept is indistinct (11). Fatigue is usually measured by questionnaires, but when using questionnaires, the results often interfere with other psychological conditions, e.g. depression (12), sleep disturbances (13), or pain (14). As depression and sleep disorders are common in patients with CP it is difficult to determine whether the fatigue seen in these patients is primarily related to pain or whether it is secondary to depression (15), which incites a general attitude that fatigue among patients with CP is due to sleep disorders and depression (16). Still, fatigue can, but need not, be linked to impaired cognitive functioning (17). Cognitive aspects of fatigue, cognitive fatigue, is a state where a cognitively demanding task give rise to a mental/subjective experience of fatigue and has often been used synonymously with mental fatigue (18). However, it is still a subjective experience. Some cognitive aspects of fatigue can also be measured on a functional level as cognitive fatigability, defined as performance decline on attention-demanding tasks (19-21).

It is well established from studies with self-reported data and cognitive tests that patients with CP are severely bothered by cognitive impairment (22-24), not least patients with generalized pain (25). Also, there are several studies showing an association between pain and deficits in attention, memory, processing speed, and executive function (22, 26). It is important to separate self-reported symptoms from functional disabilities on objective tests since, as noted above, self-reports can be influenced by psychological well-being (27).

Impaired cognitive ability in CP has been explained either by "Limited resources" i.e., that the pain competes for limited attentional resources in the brain (25, 28), or by neuroplastic changes, where impaired cognitive ability in patients with CP is supposed to be caused by neurodegenerative processes. In CP the central nervous system is affected by an imbalance in the brain's pathway system, which leads to plastic changes in the brain (29). In particular, pain activates areas of the brain

For peer review only

For peer review only - http://bmjopen.bmj.com/site/about/guidelines.xhtml

that are important for attentional functions, which may explain why many patients with CP experience attention deficits (22).

In patients with acquired brain injuries and other neurological conditions, self-rated fatigue has been associated with impaired attention (30) and slower processing speed (31). Fatigability in cognitively demanding tasks has been shown in patients with mild traumatic brain injury (32), multiple sclerosis (33), and hormonal conditions (34, 35), thus constituting an objective measure of cognitive fatigue. In acquired brain injuries and other neurological conditions cognitive fatigue has been associated with dysfunctions in the cortico-striatal networks in the basal ganglia (36-39). The relation between fatigue and attention deficits in CP has not yet been pinpointed in the literature, and cognitive fatigability has, to our knowledge, not been studied in patients with CP. It cannot be ruled out that fatigue in CP and neurological conditions might share underlying mechanisms, and that objectively measured cognitive fatigability could be an objective marker of cognitive fatigue also in CP.

As fatigue is an indistinctive complaint with multifactorial origin the importance of a standardized taxonomy has been emphasized to clarify and improve the assessment and reporting of fatigue (11). Correlations between depression and self-rated fatigue are seen in several studies regardless of the underlying medical condition (12, 40, 41). Finding methods to capture fatigue without the result being affected by depression is important to disentangle other possible underlying causes of fatigue and thereby enable effective treatment. In previous studies on patients with brain injuries, cognitive fatigability has not been affected by depression (42, 43). By combining self-rating measures and fatigue measures not related to depression but rather with functional networks in the brain (32) or with results on cognitive tests (42), a clearer picture of possible underlying causes of fatigue can be obtained and in the long run also what treatment should be recommended.

Functional MRI (fMRI) may reflect increased or decreased neuronal activity in the brain as changes in neuronal activity are linked to changes in regional blood flow (rCBF) and blood oxygenation levels (BOLD). With different fMRI techniques, it is possible to study whether discrete cerebral dysfunctions can be linked to self-perceived fatigue and cognitive fatigue/fatigability on a group level. Fatigue among patients with brain injuries has been linked to specific networks for attention in the brain, involving fronto-subcortical regions (44-46). Möller and Nordin have shown that fatigue in mild traumatic brain injury is related to altered connectivity in the brain (46) and that different neural networks are related to subjective compared to objective measures of fatigue (32). Whether similar patterns can also be seen in patients with pain and fatigue needs to be investigated. Connectivity changes have also been seen in CP, and patients with different pain conditions show connectivity

For peer review only - http://bmjopen.bmj.com/site/about/guidelines.xhtml

For peer review only

changes (47). These studies have not included fatigue and fatigability and have not used task-fMRI in the scanner.

Since chronic low-grade inflammation is thought to play a major role in the onset of chronic pain and some previous studies have shown elevated levels of inflammatory biomarkers in patients with chronic generalized pain and neuropathic pain (48-51), the immunological biomarkers in the present study are being investigated to improve knowledge on underlying mechanisms which may lead to future potential diagnostic markers. A low-grade systemic inflammation may be related to fatigue and cognition as previous studies have suggested an upregulation or dysregulation of components in the immune system in patients with chronic fatigue syndrome (52, 53) and Wåhlén et al have reported significant correlations between immunity proteins and psychological distress in patients with fibromyalgia (54). As an answer to the activation of the immune system activation in central immunocompetent cells such as microglia and astrocytes are initiated, resulting in a production of pro-inflammatory cytokines in the CNS that promote a change in behavior response characterized by decreased mood, increased anxiety, pain sensitivity, etc. Previous studies have investigated single inflammatory markers and reported inconsistent results.

There is thus a need for increased knowledge about the association between CP, cognition, and fatigue and possible underlying pathophysiological mechanisms. To improve diagnostic and prognostic models we aim to investigate the co-expression profile of inflammatory biomarkers and by applying network analysis we aim to discover cellular pathways that might be activated in cognitive fatigue.

OBJECTIVES

The primary aim of the study is to investigate the presence of self-rated mental fatigue, objectively measured cognitive fatigability, and executive functions and how these are related to other cognitive functions, pain-related factors, inflammatory biomarkers, and brain connectivity in patients with CP.

1. Do patients with CP show more pronounced self-rated mental fatigue and cognitive fatigue compared to healthy controls?
2. Are the different fatigue measures related to cognitive functions such as process speed and attention functions?

For peer review only - http://bmjopen.bmj.com/site/about/guidelines.xhtml

3. What is the relationship between a) cognitive impairments, as measured by several different cognitive tests, b) mental fatigue as measured by Multidimensional fatigue inventory-20 and VAS-f, or c) cognitive fatigability as measured by WAIS-III Coding and
    a. the duration of pain
    b. generalization or intensity of pain

   and are they affected by covariates, such as sleep disorders and degree of depression/anxiety?
4. Is there an association between immunological biomarkers and a) cognitive functions, b) mental fatigue, and c) cognitive fatigability?
5. Does the connectivity in the brain of female patients with CP differ from healthy controls at rest as well as during activity while performing a vigilance task?
6. Is there an association between connectivity in the brain and
    a. immunological biomarkers
    b. fatigability and
    c. results on neuropsychological tests?

For peer review only

Methods and analysis

Study design

This is a cross-sectional explorative case-control study. STROBE-reporting guidelines will be used (55).

Study setting

The Unit of Pain Rehabilitation at the Department of Rehabilitation Medicine at Danderyd University Hospital, Stockholm and the Department of Pain Rehabilitation, Pain Center, Umeå University Hospital, Umeå from September 2018 to October 2022. Data collection is now complete but no analyses have been completed and reported.

Participants

Patients for neuropsychological assessment

Two hundred outpatients between 18 - 50 years with chronic pain (CP) from the Unit of Pain Rehabilitation at the Department of Rehabilitation Medicine at Danderyd University Hospital or from the Department of Pain Rehabilitation, Pain Center at Umeå University Hospital will be included. There will be no stratification of age, but the majority of the patients will be women as they are overrepresented among those receiving rehabilitation for CP. In the analyses, the patients are compared with 36 healthy controls.

For peer review only - http://bmjopen.bmj.com/site/about/guidelines.xhtml

Please see the flowchart for an overview of the study (Figure 1).

Healthy controls

Thirty-six healthy controls will be primarily used for comparison of the results from the fMRI examination and the blood sampling. They will be matched at a group level in terms of gender, age, and educational level. They will be recruited among hospital staff or their friends. For those who only undergo neuropsychological assessment and blood sampling a token of SEK 300 will be offered and for those who do the fMRI and blood sampling a token of SEK 1000 will be offered.

Participants for fMRI

Twenty-four (24) patients, women only, between 18 – 45 years with CP and who have undergone a neuropsychological assessment at the Department of Pain Rehabilitation, Pain Center at Umeå University Hospital will also undergo an fMRI investigation. All patients fulfilling the criteria for participation in the fMRI study from Umeå the study site are offered to participate in fMRI scanning and blood sampling, until the participation rate goal (24) is reached. They will be compared with 22 female controls from the healthy control group.

Participants for blood sampling

Of the included participants 36 patients and 36 controls will undergo blood sampling for immunological markers. All participants undergoing fMRI will also be eligible for blood sampling, as stated above. In addition, 14 patients from the Danderyd site will be consecutively recruited for the blood-sampling part of the study.

Eligibility criteria

Inclusion criteria

- disabling musculoskeletal pain for >3 months
- aged 18 - 50 years for neuropsychological assessment and 18-45 for fMRI examination
- referred to the Department of Rehabilitation Medicine at Danderyd University Hospital or to the Department of Pain Rehabilitation, Pain Center, Umeå University Hospital

Exclusion criteria

- traumatic brain injury (including concussion)
- extensive psychiatric problems or substance abuse

For peer review only

For peer review only - http://bmjopen.bmj.com/site/about/guidelines.xhtml

- previously congenital or acquired brain injury and/or does not live in their own home and who needs support in everyday life
- lack of knowledge of the Swedish language
- progressive disease
- drugs with a strong sedative effect
- pregnancy
- for fMRI examination, patients with metal objects implanted in the body or metal chips in body parts (e.g. after eye damage) are excluded. Patients are also excluded if they are afraid of confined spaces or if they are left-handed.

Measurements

Background information from the Swedish Quality Register for Pain Rehabilitation (SQRP) (http://www.ucr.uu.se/nrs/). Information about medication, possible trauma, and background information and degree of sick leave will be obtained from the medical record.

Questionnaires from SQRP

- EuroQoL five dimensions questionnaire (EQ5D) (56)
- Hospital Anxiety and Depression Scale (HADS) (57)
- Insomnia Severity Index (ISI) (58)
- Multidimensional Pain Inventory (MPI) (59).
- Screening on physical activity by the National Board of Health and Welfare (60)

Other questionnaires

- Visual analog scale on fatigue (VAS-f) before and after neuropsychological assessment (32)
- Visual analog scale on fatigue (VAS-f) and pain before and after the fMRI scanning.
- Multidimensional Fatigue Inventory-20 (MFI-20) (61)
- Pain level, experienced during the neuropsychological examination, will be rated (0-10) at the end of the session.

Neuropsychological tests

- Wechsler Adult Intelligence Scale-III (WAIS-III) Digit-Symbol-Coding (Coding) measures psychomotor speed and incidental memory. The higher values the better results (62). The Coding subtest from WAIS-III will be used instead of WAIS-IV as the former has been validated for measuring fatigability (34, 43)
- WAIS-IV Matrix reasoning measures logical reasoning and premorbid cognitive level. The higher values the better results. (63)

For peer review only - http://bmjopen.bmj.com/site/about/guidelines.xhtml

- WAIS-IV Digit Span measures verbal attention span and working memory. The higher values the better results. (64, 65).
- Delis-Kaplan Executive Function Scale (D-KEFS) Color-Word Test and Word Fluency Test measure different aspects of executive functioning. For the Color-Word test lower values represent better results and for the Word Fluency Test, higher values represent better results (66).
- Ruff 2 & 7 measures automatic visual scanning and complex selective attention and processing speed. The higher values the better results (67).
- MapCog Spectra is a newly developed attention test that is administered via an iPad. The MapCog test records and analyzes the occurrence and frequency of attentional lapses and compares these to standardized norms. The standardization is based on a healthy reference group, n=313, aged 8 - 87 years. The test has a sensitivity of 95.8% and a specificity of 96% for identifying individuals with attention deficit disorder. The test is easy to perform (5-10 minutes), the data processing is automatic, the results are objective and there are no learning effects. The lower values the better results (68). We also measure fatiguability by comparing the results at the end of the test with the beginning of the test.

For all tests mainly raw score scores will be used in the analysis.

Primary outcome variables

The outcome variables are summarized in table 1.

Table 1. Outcome measures

Primary outcome measures	Description
**1. Fatigability from WAIS-III Coding**	The subject must fill in the blank spaces with the symbol which is paired to the number during 120 seconds. Cognitive fatigue is assessed by subtracting the number of digits produced in the first 30 seconds from the number of digits produced in the last 30 seconds during the full 120-second period. A non-ascending score (< 0) is considered an indicator of cognitive fatigue. Both the total value and the difference between the production between 0-30 seconds and 91-120 seconds are measured, and a dichotomized variable (non-ascending value) will be used.
**2. D-KEFS Color-Word Test**	Inhibition of over-learned verbal responses. The test has four conditions: 1) naming colors (red, blue, or green, 2) reading color words printed in black, 3) naming the color of the words red, blue, or green written in a different color than is the written word, which means inhibition of an over-learned function of reading the word; 4) repeatedly switching between naming colors and reading out the printed words as quickly as possible, while at the same time, the person needs to keep track of clues that indicate rule change. Contrast scores are used to examine the performance of the more complex tasks 3 and 4 and the basic tasks 1 and 2. Faster time represents a better result.

For peer review only - http://bmjopen.bmj.com/site/about/guidelines.xhtml

**3. Fatigability on e-prime vigilance task in the fMRI scanner**	The participants are instructed to push a button as quickly as they can when a set of four zeroes appears in a red rectangle and do nothing if other numbers appear. After each response, visual feedback of the reaction time is displayed. If the participant reacts to a false stimulus or if the response time exceeds 1 second the feedback "false answer" or "no answer" is displayed. The stimuli are presented at random intervals. The results are divided into quintiles and the mean reaction time is calculated for each quintile.
**4. Task-fMRI**	BOLD signal changes during the e-prime vigilance task.
**5. Resting-state fMRI**	Changes in functional connectivity after performance of the e-prime vigilance task.
**6. Inflammatory markers**	Omics analyses will be used in this study. Omics studies are mainly exploratory and hypothesis-generating, i.e., which proteins, metabolites, and lipoproteins are identified cannot be determined in advance. Exploratory targeted analyses using panels of cytokines & chemokines and neuroinflammation will be used in combination with omics.
**Secondary outcome measures**	**Description**
**1. MFI-20**	The MFI-20 consists of five scales, based on different modes of expressing fatigue. Each scale contains four items for which the person has to indicate on a seven-point scale to what extent the particular statement applies best. 'General fatigue' includes general statements concerning a person's functioning. 'Physical fatigue' refers to the physical sensation related to the feeling of tiredness. 'Reduced activities' measures reduction in activities and 'Reduced motivation' is lack of motivation. 'Mental fatigue' measures cognitive symptoms related to fatigue. Some sentences are inverted and need to be rescored. On each scale, higher values represent higher levels of fatigue.
**2. Visual Analog Scale of Fatigue**	Measurement of self-rated current fatigue level. Ranges from 0 (corresponding to no fatigue) to 100 millimeters (corresponding to the worst possible fatigue).
**3. D-KEFS Word Fluency Test**	The test measures expressive language skills, initiative, and working memory and consists of three different conditions: verbal phonological fluency (1) where the test person for 60 seconds produces as many unique words as possible that begin with a given letter; category fluency (2) where the test person for 60 seconds per category produces as many unique words as possible in two given semantic categories and; category change fluency (3) where the test person produces as many unique words as possible in 60 seconds and switches between two specified semantic categories every other time. The more words, the better performance.
**4. WAIS-IV Digit Span forward and backward repetition (Attention span and working memory)**	The test person must repeat numbers that the leader reads out. The number of digits is increased by one unit every two times. The test person repeats the numbers in the same order (forward repetition) or reverse order (backward repetition). Forward repetition measures auditory attention span and short-term memory, while backward repetition measures auditory working memory. Both the total number of digits and the difference between forward and backward repetition are measured.

For peer review only - http://bmjopen.bmj.com/site/about/guidelines.xhtml

Abbreviations: BOLD – Blood-Oxygen-Level Dependent; D-KEFS - Delis-Kaplan Executive Function Scale; Coding – Digit-Symbol Coding Test; fMRI - Functional magnetic resonance imaging; MFI - Multidimensional Fatigue Inventory; WAIS– Wechsler Adult Intelligence Scale.

DATA COLLECTION AND MANAGEMENT

RECRUITMENT

The pain rehabilitation unit at the Department of Rehabilitation Medicine at Danderyd University Hospital and the Department of Pain Rehabilitation, Pain Center at Umeå University Hospital offer team assessment and evidence-based team-based multimodal rehabilitation for patients with complex CP (>3 months). To receive rehabilitation at the clinics, a referral including a pain assessment is required. Information about the study is sent to the referred patients at the same time as the call for the initial assessment.

At the time of the team assessment, the patient is asked orally by the team nurse if the testing psychologist may contact the patient to inform them about the study. The patient's consent is logged, and a log is made of the number who say yes and the number and gender and age of those who say no. No personal data other than gender and age will be registered for those who say no. The purpose of the log is only to be able to assess generalizability based on the proportion who have agreed to participate in the study.

The testing psychologist then calls the patients and inform them about the study and if the patient agrees, an appointment is made for neuropsychological assessment. In connection with the assessment, the psychologist ensures that they have understood the information about the study and that participation is voluntary, inquires about whether they have further questions, and collects the signed consent form. Female patients who are investigated in Umeå are asked if they want to participate in the fMRI study. The patients are assessed by an independent psychologist, who does not treat the patients clinically.

The study patients will be compared with healthy controls. Healthy controls will be recruited through advertising among hospital staff. Matching on a group level will be done regarding age, gender, and level of education.

IMAGING

In a 3-tesla MR scanner, conventional anatomical MRI sequences will be analyzed as well as sequences for detailed anatomical assessment. We use an established measurement paradigm that has previously been used on healthy subjects (69) and patients with mild traumatic brain injury (70).

All participants are examined with fMRI during a 20-minute vigilance (reaction time) task (e-prime), where fatigue is measured by recording mean reaction time/quintile. BOLD resting-state fMRI

For peer review only

For peer review only - http://bmjopen.bmj.com/site/about/guidelines.xhtml

examination is performed before and after the task. In addition, the MRI protocol will include standard clinical protocols that include high-resolution T1-weighted sequences and high-resolution T2-weighted Scan (FLAIR). All structural images are reviewed by a radiologist to screen for incidental findings within the brain. The investigation is non-invasive and takes about 60 minutes.

Blood sampling

Venous blood samples (10-20 ml) are collected in P100 tubes from BD diagnostic, containing a protease inhibitor cocktail that prevents protein cleavage/degradation from each subject. The blood samples are centrifuged to remove red blood cells. The plasma fraction is transferred to a new tube, portioned, and stored at -86 ° C until analysis at the PAINOMICS lab in Linköping, Sweden.

Plasma samples will be analyzed using antibody-based methods such as multiplex technology (Meso Scale Diagnostics, Rockville, MD). Instrument, MSD Technology platform, which is a multiplex instrument, which can analyze up to 92 different substances (cytokines, chemokines, growth factors, and metabolic proteins) in one go. Omics analysis consisting of proteomics, metabolomics, and lipoprotein profiling will be used to identify other inflammatory markers than those analyzed by the antibody-based (targeted analysis) method.

Data management

All data material will be recorded with a participant ID and will be unidentifiable for those who do not have access to the code key. In the statistical processing, the participants will be coded. The code key is stored separately from the data and only pseudonymized data will be shared between the two sites. De-identified data will be electronically stored on the server at the two sites and will be deleted 10 years after the project has ended. The final dataset will be available to researchers actively contributing to statistical analyses and publications. Data entry will be controlled by initial exploratory analyses, including range checks, to promote data quality. Regarding biological specimens, the samples of blood will be centrifuged and plasma will be aliquoted and stored at the Linköping University Hospital, with special notification to the biobank. The samples will be blinded before analysis. This study has an exploratory character as we use proteomic methods, and therefore samples (that are not thawed) will be stored for future analysis according to approved ethical application.

Sample size

Power calculation on the primary fatigue measure, the WAIS-III Coding Test: For a mean difference between patients and controls of 2.5, and standard deviation of 3.0, and an alpha level of 5% (two-

For peer review only

For peer review only - http://bmjopen.bmj.com/site/about/guidelines.xhtml

sided) and strength of 80%, a group of 24 participants in each group is a sufficiently large sample (71). This size also holds for the fMRI sub-study as an earlier study on mild traumatic brain injury has shown that 10 patients in each group were sufficient (46). The biomarkers data will be analyzed using multivariate data analysis (MVDA). Algorithms for sample size calculations using MVDA do not exist. MVDA is designed to handle a few subjects, low-to-variable ratios, and multiple intercorrelated variables. Based on our previous biomarkers study (49, 72), the sample size in this study will be sufficient to detect a difference between groups”**.**

Confidentiality

Information on participants will be handled by healthcare professionals adhering to Swedish Law ensuring confidentiality and data protection by coding individual participants’ collected data. Results and data will be presented at a group level in publications, rendering the identification of individual patients impossible. All data will be stored in accordance with the General Data Protection Regulation (GDPR). The data for participants from the Stockholm area will be stored in a password-protected project server at Danderyd University Hospital and for patients from the Umeå region at Umeå University. Data will not be accessed by unauthorized persons.

Data analysis

For all study questions, descriptive statistics will be used to depict demographics, injury characteristics, results on neuropsychological tests, and psychological screening instruments. Multiple linear and logistic regression will be used to detect any interaction effects. Parametric methods such as independent t-test, One-sample t-test (comparing with standardized test norms), Pearson correlation, and multiple linear regression) will be used when data is normally distributed and non-parametric methods (Man-Whitney U-test, Spearman correlation, Chi2, logistic regression) when data is skewed or on interval level or lower. Missing data will be listed as data loss. The significance level will be set to <.05 (two-tailed).

Concerning study question 4, data from omics and targeted analysis will be analyzed using advanced multivariate data analysis using SIMCA-P+. This is the recommended method in omics since it accounts for multicollinearity problems and missing data when the number of variables exceeds the number of observations (73). MVDA will be used for the analysis of the biomarkers. Principal component analysis (PCA), Orthogonal partial least squares- discriminant analysis (OPLS-DA), and OPLS regression will be applied for analysis of the biomarkers and their ability to differentiate between groups and their correlation with clinical characteristics and outcomes. PCA is an unsupervised analysis that reduces the

For peer review only

For peer review only - http://bmjopen.bmj.com/site/about/guidelines.xhtml

dimensionality of data and extracts and displays systematic variation in the data matrix. Outliers will be identified using score plots in combination with Hotelling's T2 test (the distance from the origin in the model plane for each selected observation) and DModX (the distance of the observation to the X model plane). The values that are larger than the 95% confidence limit are considered suspicious and the values larger than the 99% limit should be considered strong outliers (74).

OPLS-DA will be used to identify biomarkers responsible for group separation. The OPLS-DA model reveals variables as loadings and the higher the value of a loading the more important it is for the model, e.g., group separation. This can be measured as a Variable influence on projection (VIP) values. The p(corr) is the loading of each variable scaled as a correlation coefficient (ranging from -1 to +1). An absolute p(corr) of >0.4-0.5 and VIP > 1.0 are considered significant (73). Another model diagnosis parameters that are considered are R2 value- which describes the "goodness of fit", i.e., the fraction of the sum of squares of all the variables explained by a principal component. The Q2 value describes the "goodness of prediction", i.e., the fraction of the total variation of the variables that can be predicted by a principal component using a cross-validation method (74) and cross-validated analysis of variance (CV-ANOVA). The R2 must not differ more than 0.2-0.3 compared to Q2 and CV-ANOVA< 0.05 is considered a significant model.

Regarding study question 5 analysis of fMRI data will be done with special software for MRI analysis, developed at Oxford University (http://www.fmrib.ox.ac.uk/fsl/). The resting state data will be analyzed using FSL tools such as MELODIC to identify spatial and temporal components by independent component analysis at the group level. All relevant preprocessing steps will be performed, such as motion correction, registration to a standard template, and filtering of low-frequency drifts. Group differences between patients and controls will be analyzed using FSL dual regression. The fMRI data will be analyzed using both MELODIC and FEAT which is an FSL tool based on general linear modeling performing multiple regression on the fMRI data and the functional paradigm.

For study question 6 multiple linear and logistic regression will be used to detect any interaction effects.

PLANS FOR COMMUNICATING IMPORTANT PROTOCOL AMENDMENTS TO RELEVANT PARTIES

Important protocol modifications will be reported to the Ethics committee in Sweden and amendments will be made to the trial registry (Clinicaltrials.gov).

For peer review only

For peer review only - http://bmjopen.bmj.com/site/about/guidelines.xhtml

Patient and public involvement

None.

Ethics and dissemination

The study was approved by the Swedish Ethical Review Authority (2018/424-31; 2018/1235-32; 2018/2395-32; 2019-66148; 2022-02838-02). All participants are provided oral and written information by the neuropsychologists (AH and NB) and will give written informed consent to participate in the study.

Publications are planned for journals in the fields of pain, neuropsychology, and rehabilitation. Results will further be spread at relevant conferences, national and international meetings, and expert forums. The results will be shared with user organizations and its members as well as relevant policy makers.

Contributors

MCM, ML, and BMS formed the original research concept. BG, LN, NB, and AH contributed to the study design within their field of expertise. MCM and BMS will coordinate the project in Stockholm and Umeå in cooperation with NB, BG, AH, ML, and LN. NB and AH will be responsible for data collection and statistical analyses of the neuropsychological and demographic data in collaboration with MCM, ML, and BMS. LN will be responsible for fMRI analyses in collaboration with NB. BG will be responsible for the analyses of biomarkers. MCM has written the manuscript draft. All authors have contributed with important intellectual content to the manuscript. Regarding upcoming manuscripts, we will use authorship eligibility guidelines. We will not use professional writers.

Funding

This study is supported by Promobilia Foundation, grant number [A22056], the research and development fund granted by the County Council of Västerbotten [RV-928951; RV-939460], and through a regional agreement between Umeå University and Västerbotten County Council (ALF) [RV-930845; RV-940388; RV-967132]. The Department of Rehabilitation Medicine and the Karolinska Institutet, Department of Clinical Sciences, Danderyd University Hospital have also provided valuable support for the study to be feasible. The funders have no vested interest in the study and have not influenced the design.

For peer review only - http://bmjopen.bmj.com/site/about/guidelines.xhtml

COMPETING INTERESTS

None declared.

PATIENT CONSENT FOR PUBLICATION

Not applicable.

REFERENCES

1. Ng SK, Urquhart DM, Fitzgerald PB, Cicuttini FM, Hussain SM, Fitzgibbon BM. The Relationship between Structural and Functional Brain Changes and Altered Emotion and Cognition in Chronic Low Back Pain: A Systematic Review of MRI and fMRI Studies. Clin J Pain. 2017.
2. Flodin P, Martinsen S, Mannerkorpi K, Lofgren M, Bileviciute-Ljungar I, Kosek E, et al. Normalization of aberrant resting state functional connectivity in fibromyalgia patients following a three month physical exercise therapy. Neuroimage Clin. 2015;9:134-9.
3. Marchand S. The physiology of pain mechanisms: from the periphery to the brain. Rheum Dis Clin North Am. 2008;34(2):285-309.
4. Creavin ST, Dunn KM, Mallen CD, Nijrolder I, van der Windt DA. Co-occurrence and associations of pain and fatigue in a community sample of Dutch adults. Eur J Pain. 2010;14(3):327-34.
5. Hart RP, Martelli MF, Zasler ND. Chronic pain and neuropsychological functioning. Neuropsychol Rev. 2000;10(3):131-49.
6. Wolfe F, Hawley DJ, Wilson K. The prevalence and meaning of fatigue in rheumatic disease. J Rheumatol. 1996;23(8):1407-17.
7. Bjurstrom MF, Irwin MR. Polysomnographic characteristics in nonmalignant chronic pain populations: A review of controlled studies. Sleep Med Rev. 2016;26:74-86.
8. Fishbain DA, Cole B, Cutler RB, Lewis J, Rosomoff HL, Fosomoff RS. Is pain fatiguing? A structured evidence-based review. Pain Med. 2003;4(1):51-62.
9. Louati K, Berenbaum F. Fatigue in chronic inflammation - a link to pain pathways. Arthritis Res Ther. 2015;17:254.
10. Arnold LM, Crofford LJ, Mease PJ, Burgess SM, Palmer SC, Abetz L, et al. Patient perspectives on the impact of fibromyalgia. Patient Educ Couns. 2008;73(1):114-20.
11. Kluger BM, Krupp LB, Enoka RM. Fatigue and fatigability in neurologic illnesses: proposal for a unified taxonomy. Neurology. 2013;80(4):409-16.
12. Arnold LM. Understanding fatigue in major depressive disorder and other medical disorders. Psychosomatics. 2008;49(3):185-90.
13. Akerstedt T, Knutsson A, Westerholm P, Theorell T, Alfredsson L, Kecklund G. Mental fatigue, work and sleep. J Psychosom Res. 2004;57(5):427-33.
14. Snekkevik H, Eriksen HR, Tangen T, Chalder T, Reme SE. Fatigue and depression in sick-listed chronic low back pain patients. Pain Med. 2014;15(7):1163-70.
15. Mease P, Arnold LM, Bennett R, Boonen A, Buskila D, Carville S, et al. Fibromyalgia syndrome. J Rheumatol. 2007;34(6):1415-25.
16. Kurtze N, Svebak S. Fatigue and patterns of pain in fibromyalgia: correlations with anxiety, depression and co-morbidity in a female county sample. Br J Med Psychol. 2001;74(Pt 4):523-37.
17. Johnson SK, Lange G, DeLuca J, Korn LR, Natelson B. The effects of fatigue on neuropsychological performance in patients with chronic fatigue syndrome, multiple sclerosis, and depression. Appl Neuropsychol. 1997;4(3):145-53.
18. Wylie GR, Flashman LA. Understanding the interplay between mild traumatic brain injury and cognitive fatigue: models and treatments. Concussion (London, England). 2017;2(4):Cnc50.
19. Schwid SR, Tyler CM, Scheid EA, Weinstein A, Goodman AD, McDermott MP. Cognitive fatigue during a test requiring sustained attention: a pilot study. Mult Scler. 2003;9(5):503-8.
20. Bryant D, Chiaravalloti ND, DeLuca J. Objective Measurement of Cognitive Fatigue in Multiple Sclerosis. Rehabilitation psychology. 2004;49(2):114.

For peer review only - http://bmjopen.bmj.com/site/about/guidelines.xhtml

21. Walker LAS, Lindsay-Brown AP, Berard JA. Cognitive Fatigability Interventions in Neurological Conditions: A Systematic Review. Neurology and therapy. 2019;8(2):251-71.
22. Moriarty O, McGuire BE, Finn DP. The effect of pain on cognitive function: a review of clinical and preclinical research. Prog Neurobiol. 2011;93(3):385-404.
23. Ferreira Kdos S, Oliver GZ, Thomaz DC, Teixeira CT, Foss MP. Cognitive deficits in chronic pain patients, in a brief screening test, are independent of comorbidities and medication use. Arq Neuropsiquiatr. 2016;74(5):361-6.
24. Rathbone M, Parkinson W, Rehman Y, Jiang S, Bhandari M, Kumbhare D. Magnitude and variability of effect sizes for the associations between chronic pain and cognitive test performances: a meta-analysis. Br J Pain. 2016;10(3):141-55.
25. Grisart J, Van der Linden M, Masquelier E. Controlled processes and automaticity in memory functioning in fibromyalgia patients: relation with emotional distress and hypervigilance. J Clin Exp Neuropsychol. 2002;24(8):994-1009.
26. Higgins DM, Martin AM, Baker DG, Vasterling JJ, Risbrough V. The Relationship Between Chronic Pain and Neurocognitive Function: A Systematic Review. Clin J Pain. 2018;34(3):262-75.
27. Lundin A, de Boussard C, Edman G, Borg J. Symptoms and disability until 3 months after mild TBI. Brain Inj. 2006;20(8):799-806.
28. Legrain V, Damme SV, Eccleston C, Davis KD, Seminowicz DA, Crombez G. A neurocognitive model of attention to pain: behavioral and neuroimaging evidence. Pain. 2009;144(3):230-2.
29. Ng K, Sin , Urquhart M, Donna , Fitzgerald B, Paul , Cicuttini M, Flavia , Hussain M, Sultana , Fitzgibbon M, Bernadette. The Relationship Between Structural and Functional Brain Changes and Altered Emotion and Cognition in Chronic Low Back Pain Brain Changes: A Systematic Review of MRI and fMRI Studies. The Clinical Journal of Pain. 2018;34(3):237-61.
30. Ziino C, Ponsford J. Measurement and prediction of subjective fatigue following traumatic brain injury. J Int Neuropsychol Soc. 2005;11(4):416-25.
31. Johansson B, Berglund P, Rönnbäck L. Mental fatigue and impaired information processing after mild and moderate traumatic brain injury. Brain Inj. 2009;23(13-14):1027-40.
32. Möller MC, Nordin LE, Bartfai A, Julin P, Li TQ. Fatigue and Cognitive Fatigability in Mild Traumatic Brain Injury are Correlated with Altered Neural Activity during Vigilance Test Performance. Front Neurol. 2017;8:496.
33. Walker LA, Berard JA, Berrigan LI, Rees LM, Freedman MS. Detecting cognitive fatigue in multiple sclerosis: method matters. J Neurol Sci. 2012;316(1-2):86-92.
34. Möller MC, Bartfai A, Nygren de Boussard C, Flöter Rådestad A, Calissendorff J. High rates of fatigue in newly diagnosed Graves' disease. Fatigue: Biomedicine, Health & Behavior. 2014;2(3):153-62.
35. Möller MC, Rådestad AF, von Schoultz B, Bartfai A. Effect of estrogen and testosterone replacement therapy on cognitive fatigue. Gynecol Endocrinol. 2013;29(2):173-6.
36. Chaudhuri A, Behan PO. Fatigue and basal ganglia. J Neurol Sci. 2000;179(S 1-2):34-42.
37. DeLuca J, Genova HM, Hillary FG, Wylie G. Neural correlates of cognitive fatigue in multiple sclerosis using functional MRI. J Neurol Sci. 2008;270(1-2):28-39.
38. Kohl AD, Wylie GR, Genova HM, Hillary FG, Deluca J. The neural correlates of cognitive fatigue in traumatic brain injury using functional MRI. Brain Inj. 2009;23(5):420-32.
39. Chaudhuri A, Behan PO. Fatigue in neurological disorders. Lancet. 2004;363(9413):978-88.
40. Holmqvist A, Lindstedt MB, Moller MC. Relationship between fatigue after acquired brain injury and depression, injury localization and aetiology: An explorative study in a rehabilitation setting. J Rehabil Med. 2018;50(8):725-31.
41. van Barneveld E, Manders J, van Osch FHM, van Poll M, Visser L, van Hanegem N, et al. Depression, Anxiety, and Correlating Factors in Endometriosis: A Systematic Review and Meta-Analysis. J Womens Health (Larchmt). 2022;31(2):219-30.
42. Holmqvist A, Bartfai A, Markovic G, Möller MC. Does Intensive Training of Attention Influence Cognitive Fatigability in Patients With Acquired Brain Injury? Front Neurosci. 2021;15:656876.
43. Möller MC, Nygren de Boussard C, Oldenburg C, Bartfai A. An investigation of attention, executive, and psychomotor aspects of cognitive fatigability. J Clin Exp Neuropsychol [Internet]. 2014; 26:[1-14 pp.].
44. Cantor F. Central and peripheral fatigue: exemplified by multiple sclerosis and myasthenia gravis. Pm & R. 2010;2(5):399-405.
45. Dobryakova E, Deluca J, Genova HM, Wylie GR. Neural Correlates of Cognitive Fatigue: Cortico-Striatal Circuitry and Effort-Reward Imbalance. Journal Of The International Neuropsychological Society: JINS. 2013:1-5.

For peer review only - http://bmjopen.bmj.com/site/about/guidelines.xhtml

46. Nordin LE, Moller MC, Julin P, Bartfai A, Hashim F, Li TQ. Post mTBI fatigue is associated with abnormal brain functional connectivity. Sci Rep. 2016;6:21183.
47. Apkarian AV. Frontiers in Neuroscience. Human Brain Imaging Studies of Chronic Pain: Translational Opportunities. In: Kruger L, Light AR, editors. Translational Pain Research: From Mouse to Man. Boca Raton, FL: CRC Press/Taylor & Francis Llc.; 2010.
48. Gerdle B, Backryd E, Falkenberg T, Lundstrom E, Ghafouri B. Changes in inflammatory plasma proteins from patients with chronic pain associated with treatment in an interdisciplinary multimodal rehabilitation program - an explorative multivariate pilot study. Scandinavian journal of pain. 2019.
49. Jönsson M, Gerdle B, Ghafouri B, Bäckryd E. The inflammatory profile of cerebrospinal fluid, plasma, and saliva from patients with severe neuropathic pain and healthy controls-a pilot study. BMC Neurosci. 2021;22(1):6.
50. Olausson P, Gerdle B, Ghafouri N, Sjöström D, Blixt E, Ghafouri B. Protein alterations in women with chronic widespread pain--An explorative proteomic study of the trapezius muscle. Sci Rep. 2015;5:11894.
51. Wåhlén K, Olausson P, Carlsson A, Ghafouri N, Gerdle B, Ghafouri B. Systemic alterations in plasma proteins from women with chronic widespread pain compared to healthy controls: a proteomic study. J Pain Res. 2017;10:797-809.
52. Mandarano AH, Maya J, Giloteaux L, Peterson DL, Maynard M, Gottschalk CG, et al. Myalgic encephalomyelitis/chronic fatigue syndrome patients exhibit altered T cell metabolism and cytokine associations. J Clin Invest. 2020;130(3):1491-505.
53. Lorusso L, Mikhaylova SV, Capelli E, Ferrari D, Ngonga GK, Ricevuti G. Immunological aspects of chronic fatigue syndrome. Autoimmun Rev. 2009;8(4):287-91.
54. Wåhlén K, Ernberg M, Kosek E, Mannerkorpi K, Gerdle B, Ghafouri B. Significant correlation between plasma proteome profile and pain intensity, sensitivity, and psychological distress in women with fibromyalgia. Sci Rep. 2020;10(1):12508.
55. von Elm E, Altman DG, Egger M, Pocock SJ, Gøtzsche PC, Vandenbroucke JP. The Strengthening the Reporting of Observational Studies in Epidemiology (STROBE) statement: guidelines for reporting observational studies. Ann Intern Med. 2007;147(8):573-7.
56. Group E. EuroQol--a new facility for the measurement of health-related quality of life. Health policy (Amsterdam, Netherlands). 1990;16(3):199-208.
57. Zigmond AS, Snaith RP. The Hospital Anxiety and Depression Scale. Acta Psychiatrica Scandinavica. 1983.
58. Bastien CH, Vallieres A, Morin CM. Validation of the Insomnia Severity Index as an outcome measure for insomnia research. Sleep Med. 2001;2(4):297-307.
59. Kerns RD, Turk DC, Rudy TE. The West Haven-Yale Multidimensional Pain Inventory (WHYMPI). Pain. 1985;23(4):345-56.
60. Socialstyrelsen. Nationella riktlinjer för sjukdomsförebyggande metoder 2011. Tobaksbruk, riskbruk av alkohol, otillräcklig fysisk aktivitet och ohälsosamma matvanor. Stöd för styrning och ledning. Stockholm: Socialstyrelsen; 2011.
61. Smets EMA, Garssen B, Bonke B, De Haes JCJM. The multidimensional Fatigue Inventory (MFI) psychometric qualities of an instrument to assess fatigue. J Psychosom Res. 1995;39(3):315-25.
62. Wechsler D. Wechsler Adult Intelligence Scale - Third edition. San Antonio: Pearson; 1997.
63. Wechsler D. WAIS-IV technical and interpretive manual. San Antonio, Tex.: Psychological Corporation: Pearson; 2008.
64. Lezak MD, Howieson DB, Bigler ED, Tranel D. Neuropsychological Assessment. 5th ed. New York: Oxford University Press 2012.
65. Wechsler D. Wechsler Adult Intelligence Scale-Fourth Edition: Administration and scoring manual. San Antonio, TX: Psychological Corporation; 2008.
66. Delis DC, Kaplan E, Kramer JH. D-KEFS. Delis-Kaplan Executive Function System. San Antonio, TX: The Psychological Corporation; 2001.
67. Ruff RM, Allen CC. Ruff 2 & 7 Selective Attention Test. Lutz: Psychological Assessment Resources, Inc.; 1996.
68. Gunnarsson H, Grahn B, Agerstrom J. Impaired psychomotor ability and attention in patients with persistent pain: a cross-sectional comparative study. J Pain Res. 2016;9:825-35.
69. Lim J, Wen-chau W, Jiongjiong W, Detre JA, Dinges DF, Rao H. Imaging brain fatigue from sustained mental workload: An AS perfusion study of the time-on-task effect. Neuroimage. 2010;49:3426-35.
70. Treleaven J, Takasaki H. Characteristics of visual disturbances reported by subjects with neck pain. Man Ther. 2014;19(3):203-7.
71. Möller M. Fatigue and cognition - hormonal perspectives. Stockholm: Karolinska Institutet; 2013.

For peer review only

For peer review only - http://bmjopen.bmj.com/site/about/guidelines.xhtml

72. Ghafouri B, Edman E, Löf M, Lund E, Leinhard OD, Lundberg P, et al. Fibromyalgia in women: association of inflammatory plasma proteins, muscle blood flow, and metabolism with body mass index and pain characteristics. Pain Rep. 2022;7(6):e1042.

73. Wheelock Å M, Wheelock CE. Trials and tribulations of 'omics data analysis: assessing quality of SIMCA-based multivariate models using examples from pulmonary medicine. Mol Biosyst. 2013;9(11):2589-96.

74. Eriksson L, Johansson E, Kettaneh-Wold N, Trygg J, Wikström C, Wold S. Multi- and Megavariate Data Analysis; Part I and II. 2 ed. Umeå Umetrics AB; 2006.

FIGURE TITLE

Figure 1. Study flowchart

For peer review only

For peer review only - http://bmjopen.bmj.com/site/about/guidelines.xhtml

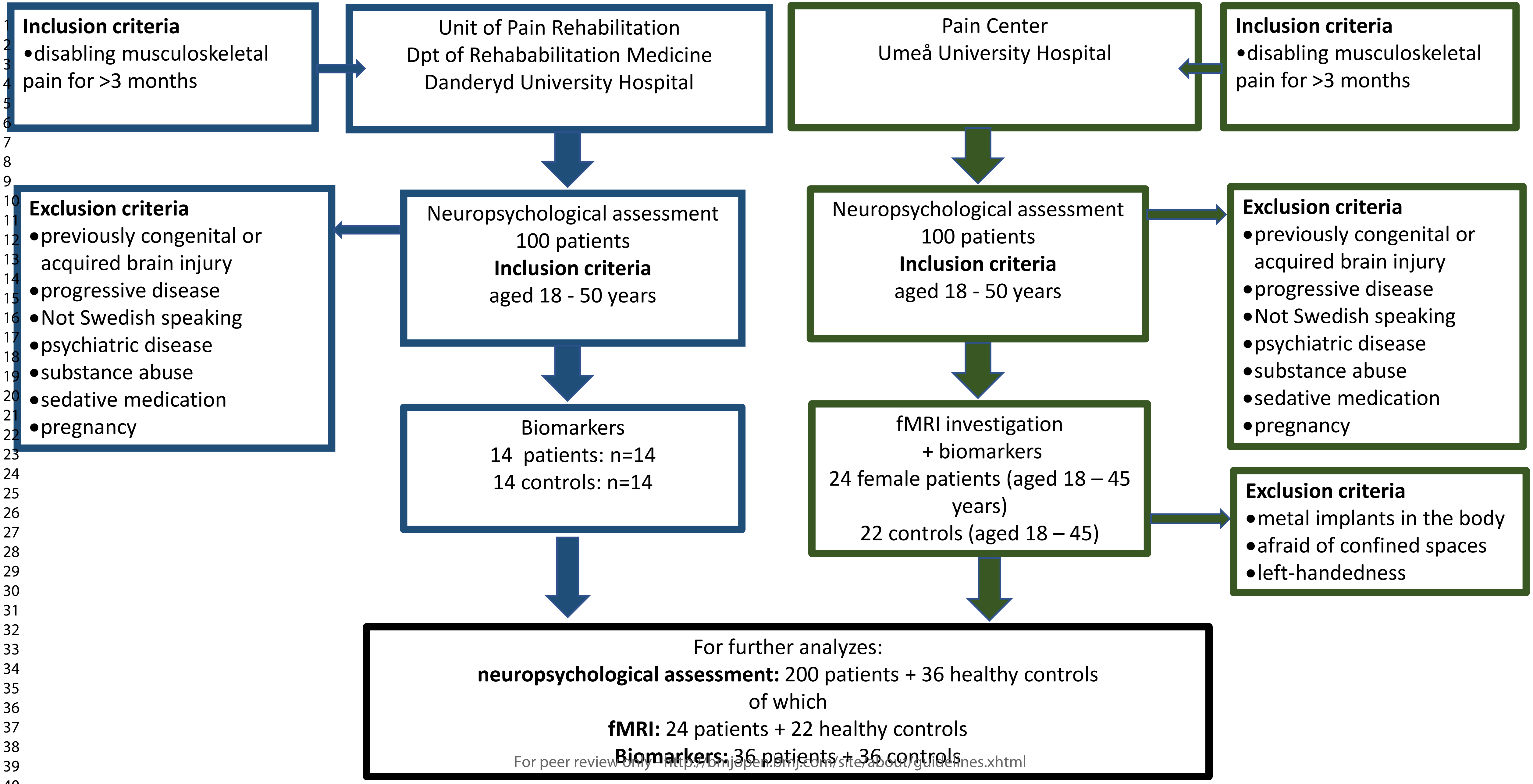
Inclusion criteria
•disabling musculoskeletal pain for >3 months
Unit of Pain Rehabilitation
Dpt of Rehababilitation Medicine
Danderyd University Hospital
Pain Center
Umeå University Hospital
Inclusion criteria
•disabling musculoskeletal pain for >3 months
Exclusion criteria
•previously congenital or acquired brain injury
•progressive disease
•Not Swedish speaking
•psychiatric disease
•substance abuse
•sedative medication
•pregnancy
Neuropsychological assessment
100 patients
Inclusion criteria
aged 18 - 50 years
Neuropsychological assessment
100 patients
Inclusion criteria
aged 18 - 50 years
Exclusion criteria
•previously congenital or acquired brain injury
•progressive disease
•Not Swedish speaking
•psychiatric disease
•substance abuse
•sedative medication
•pregnancy
Biomarkers
14 patients: n=14
14 controls: n=14
fMRI investigation
+ biomarkers
24 female patients (aged 18 – 45 years)
22 controls (aged 18 – 45)
Exclusion criteria
•metal implants in the body
•afraid of confined spaces
•left-handedness
For further analyzes:
neuropsychological assessment: 200 patients + 36 healthy controls
of which
fMRI: 24 patients + 22 healthy controls
Biomarkers: 36 patients + 36 controls

STROBE Statement—Checklist of items that should be included in reports of ***cross-sectional studies***

	Item No	Recommendation	Page No
**Title and abstract**	1	(*a*) Indicate the study's design with a commonly used term in the title or the abstract	1
		(*b*) Provide in the abstract an informative and balanced summary of what was done and what was found	1
**Introduction**			
Background/rationale	2	Explain the scientific background and rationale for the investigation being reported	4-6
Objectives	3	State specific objectives, including any prespecified hypotheses	6
**Methods**			
Study design	4	Present key elements of study design early in the paper	6
Setting	5	Describe the setting, locations, and relevant dates, including periods of recruitment, exposure, follow-up, and data collection	7
Participants	6	(*a*) Give the eligibility criteria, and the sources and methods of selection of participants	7
Variables	7	Clearly define all outcomes, exposures, predictors, potential confounders, and effect modifiers. Give diagnostic criteria, if applicable	8-10
Data sources/ measurement	8*	For each variable of interest, give sources of data and details of methods of assessment (measurement). Describe comparability of assessment methods if there is more than one group	8-10
Bias	9	Describe any efforts to address potential sources of bias	11
Study size	10	Explain how the study size was arrived at	13
Quantitative variables	11	Explain how quantitative variables were handled in the analyses. If applicable, describe which groupings were chosen and why	12
Statistical methods	12	(*a*) Describe all statistical methods, including those used to control for confounding	13
		(*b*) Describe any methods used to examine subgroups and interactions	13
		(*c*) Explain how missing data were addressed	13
		(*d*) If applicable, describe analytical methods taking account of sampling strategy	N/A
		(*e*) Describe any sensitivity analyses	12
**Results**			
Participants	13*	(a) Report numbers of individuals at each stage of study—eg numbers potentially eligible, examined for eligibility, confirmed eligible, included in the study, completing follow-up, and analysed	7
		(b) Give reasons for non-participation at each stage	8
		(c) Consider use of a flow diagram	N/A
Descriptive data	14*	(a) Give characteristics of study participants (eg demographic, clinical, social) and information on exposures and potential confounders	7-8
		(b) Indicate number of participants with missing data for each variable of interest	N/A
Outcome data	15*	Report numbers of outcome events or summary measures	N/A
Main results	16	(*a*) Give unadjusted estimates and, if applicable, confounder-adjusted estimates and their precision (eg, 95% confidence interval). Make clear which confounders were adjusted for and why they were included	N/A

For peer review only

		(*b*) Report category boundaries when continuous variables were categorized	N/A
		(*c*) If relevant, consider translating estimates of relative risk into absolute risk for a meaningful time period	N/A
Other analyses	17	Report other analyses done—eg analyses of subgroups and interactions, and sensitivity analyses	N/A
**Discussion**			
Key results	18	Summarise key results with reference to study objectives	N/A
Limitations	19	Discuss limitations of the study, taking into account sources of potential bias or imprecision. Discuss both direction and magnitude of any potential bias	N/A
Interpretation	20	Give a cautious overall interpretation of results considering objectives, limitations, multiplicity of analyses, results from similar studies, and other relevant evidence	N/A
Generalisability	21	Discuss the generalisability (external validity) of the study results	N/A
**Other information**			
Funding	22	Give the source of funding and the role of the funders for the present study and, if applicable, for the original study on which the present article is based	14

*Give information separately for exposed and unexposed groups.

Note: An Explanation and Elaboration article discusses each checklist item and gives methodological background and published examples of transparent reporting. The STROBE checklist is best used in conjunction with this article (freely available on the Web sites of PLoS Medicine at http://www.plosmedicine.org/, Annals of Internal Medicine at http://www.annals.org/, and Epidemiology at http://www.epidem.com/). Information on the STROBE Initiative is available at www.strobe-statement.org.

For peer review only

For peer review only - http://bmjopen.bmj.com/site/about/guidelines.xhtml
